# The Potential Role of Exosomal Proteins in Prostate Cancer

**DOI:** 10.3389/fonc.2022.873296

**Published:** 2022-06-07

**Authors:** Shangzhi Feng, Kecheng Lou, Xiaofeng Zou, Junrong Zou, Guoxi Zhang

**Affiliations:** ^1^ The First Clinical College, Gannan Medical University, Ganzhou, Jiangxi, China; ^2^ Department of Urology, The First Affiliated hospital of Gannan Medical University, Ganzhou, China; ^3^ Institute of Urology, The First Affiliated Hospital of Ganna Medical University, Ganzhou, China; ^4^ Department of Jiangxi Engineering Technology Research Center of Calculi Prevention, Gannan Medical University, Ganzhou, Jiangxi, China

**Keywords:** chemoresistance, exosomal proteins, prostate cancer, tumor markers, cancer treatment

## Abstract

Prostate cancer is the most prevalent malignant tumor in men across developed countries. Traditional diagnostic and therapeutic methods for this tumor have become increasingly difficult to adapt to today’s medical philosophy, thus compromising early detection, diagnosis, and treatment. Prospecting for new diagnostic markers and therapeutic targets has become a hot topic in today’s research. Notably, exosomes, small vesicles characterized by a phospholipid bilayer structure released by cells that is capable of delivering different types of cargo that target specific cells to regulate biological properties, have been extensively studied. Exosomes composition, coupled with their interactions with cells make them multifaceted regulators in cancer development. Numerous studies have described the role of prostate cancer-derived exosomal proteins in diagnosis and treatment of prostate cancer. However, so far, there is no relevant literature to systematically summarize its role in tumors, which brings obstacles to the later research of related proteins. In this review, we summarize exosomal proteins derived from prostate cancer from different sources and summarize their roles in tumor development and drug resistance.

## Introduction

Prostate cancer (PCa) is a highly prevalent and the second highest cause of cancer-related mortalities in men. Although PCa incidence is lower in Asia, relative to that in Europe and the United States, there is a continuous increasing trend (1). Currently, clinical treatment of PCa faces numerous challenges, due to its progression to Castration-resistant prostate cancer (CRPC)and a high rate of bone metastasis. Therefore, prospecting for new diagnostic and therapeutic targets is imperative to effective management of the malignancy. Previous studies have shown that novel diagnostic and therapeutic pathways, represented by exosomes, have potential for solving such problems. For example, miR-21 in PCa-derived exosomes (PCaDE) was found to inhibit apoptosis thereby promoting survival of cancer cells (2), whereas miR-423-5p was differentially expressed in PCa bone metastases a phenomenon that provided a basis for diagnosis of potential bone metastases (3). On the other hand, long non-coding RNA (lncRNA)was associated with vascular regeneration, tumor survival and metastasis, as well as tumor microenvironment (TME) establishment (4). Apart from RNA, prostate cancer-derived exosomal proteins (PCaDEPr), such as Exportin1(XPO1) which is present in all PCa cell lines exosomes, have been studied. Notably, this nuclear protein which is involved in nucleoplasmic exportation of the carry signal protein, not only plays a crucial role in the tumorigenic signaling pathway but also increases with the Gleason score (5, 6). This may be a new avenue for diagnosis and treatment of advanced PCa.

**Figure 1:**
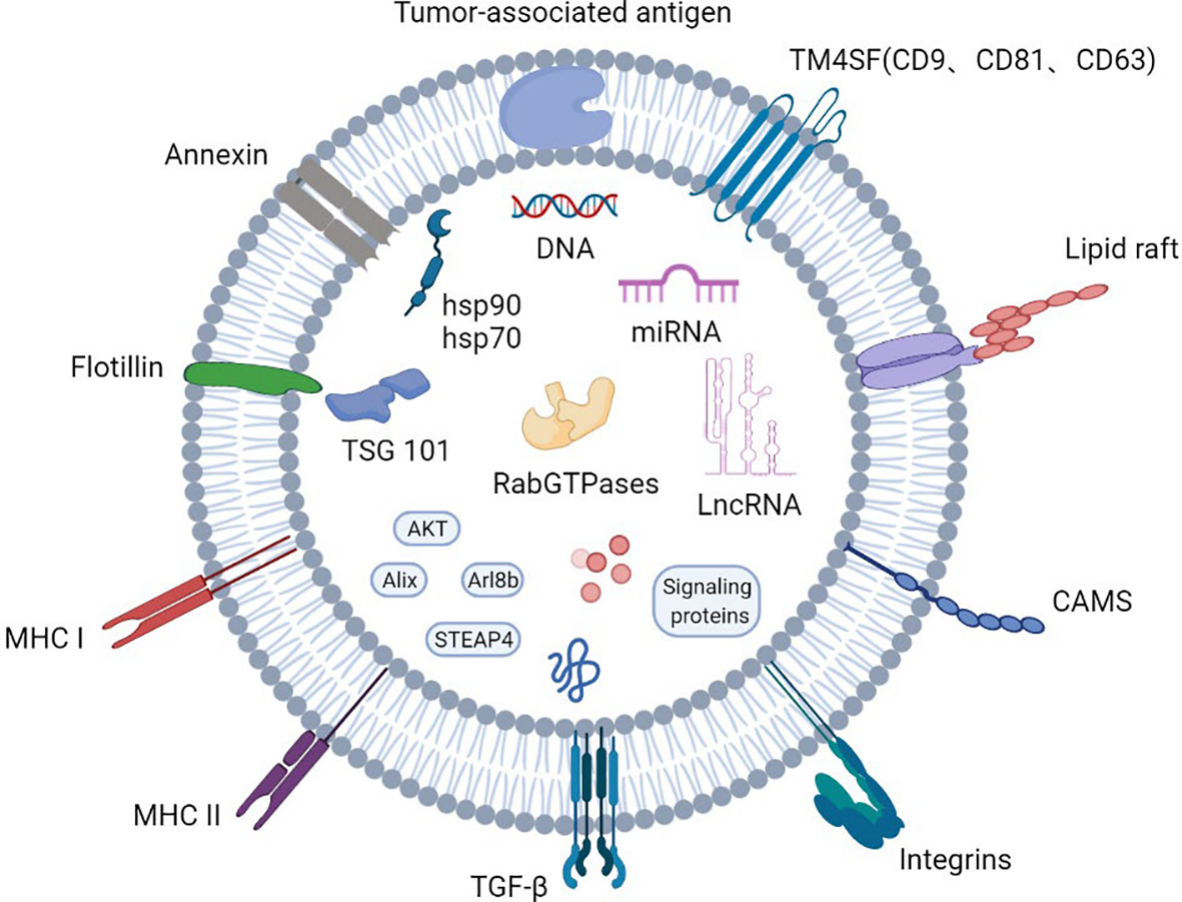
Profile of the basic structure of exosomes.

Exosomes are small membranous vesicles with a diameter of 30-150 nm that are formed by cells budding inward to form early endosomes that subsequently evolve into multi-vesicular bodies, which then fuse with the plasma membrane and are eventually released into the extracellular matrix. They participate in intercellular signaling by carrying various biomolecules such as proteins and nucleic acids, and regulate the pathophysiological processes of the organism (7). Exosomal proteins include endosomal proteins, plasma proteins and nuclear proteins. In PCa, these proteins have been shown to have a higher level of glycosylation than cellular ones (1). In addition, exosomes carry both membrane transport and fusion proteins, such as RabGTPases, Annexin, flotillins1(Flot), microvesicle-forming proteins Alix and Tsg101, as well as lipid-associated protein families including CD9, CD81, CD82and CD63 integrin proteins (8–11). Notably, the four transmembrane proteins play an important role in exosome-mediated regulation of cellular homeostasis components (**Figure 1**).

**Figure 2:**
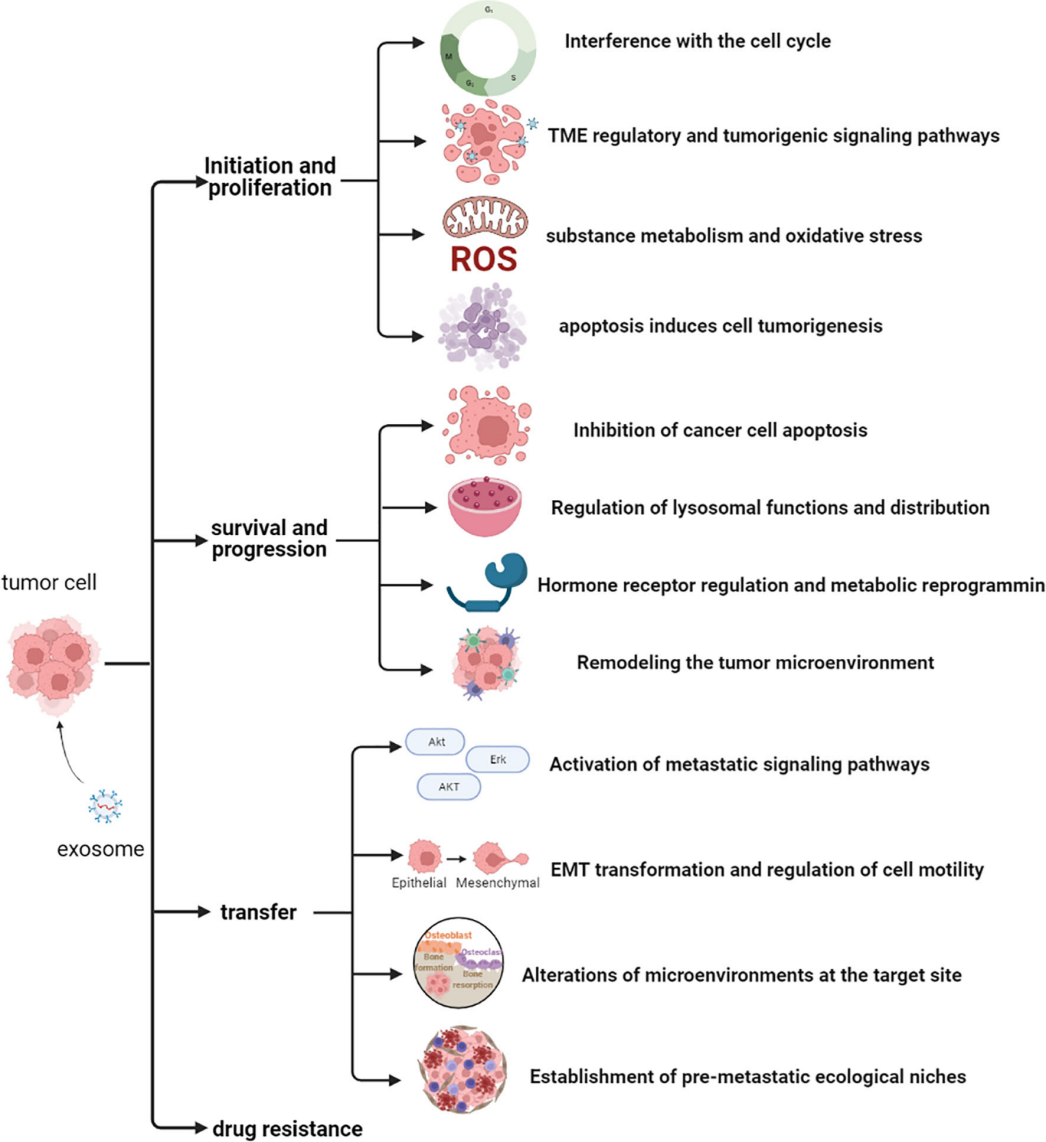
Potential mechanisms of action of exosomal proteins in prostate cancer.

**Figure 3:**
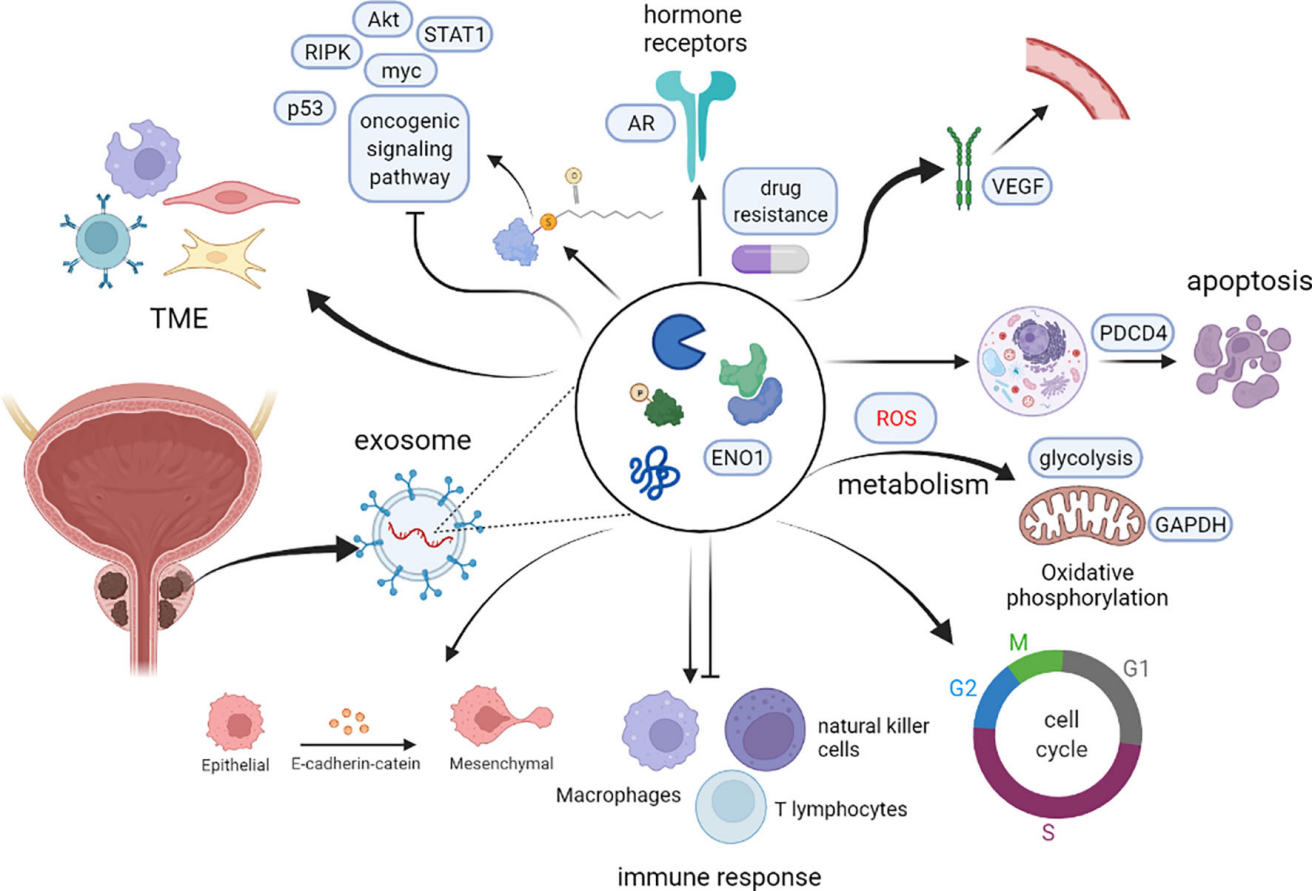
The potential role of PCaDEPrs in cancer. Exosomes originating from the tumor cell play a crucial role in tumor development. During tumor initiation, they mediate apoptosis, lipid metabolism, TME, and tumorigenic signaling, and also interfere with the cell cycle to induce cancer development. During tumor survival and progression, they regulate remodeling the tumor microenvironment, hormonal regulation and metabolic alterations, as well as lysosomal function and distribution, and inhibition of cancer cell apoptosis. During tumor metastasis, PCaDEPrs can contribute to EMT transformation, trigger microenvironment alteration, and establishment of a pre-metastatic ecological niche. Finally, they can also regulate tumor resistance to chemotherapeutic agents.

Although several studies have described the role of exosomal proteins in PCa, precise markers for PCa development have not been elucidated. Therefore, identification of the main types of PCaDEPr, coupled with elucidating the precise roles and underlying mechanisms of action for these proteins in cancer are imperative to guiding future developmental studies. Recent studies have demonstrated that exosomes proteins derived from PCa cell lines, plasma, tissues and urine are closely associated with tumor development (**Figure 2**). Therefore, understanding the roles played by these proteins, coupled with elucidating their underlying mechanisms of action in tumors will enable better targeting of these proteins for clinical treatment and improve the quality of survival of PCa patients (**Figure 3**).

## PCaDEPr From Different Sources

### PCaDEPr in the Cell Line

Findings from several exosomal proteomics and subsequent functional validation in PCa cell lines have shown that exosomal proteins secreted by the cell lines play an important role in both tumorigenesis and development (**Table 1**). The exosomal proteins secreted by PCa cell lines are relatively high in tetraspanins such as CD9, CD82, CD61, heat shock protein (HSP) family HSP90, HSP70, and integrin proteins ITGA3, ITGB1, etc., and previous studies have confirmed these Proteins may play a role in the occurrence and development of tumors. Kurozumi et al. found that knocking down ITGA3 and ITGB1 significantly downregulated phosphorylation of FAK, SRC, AKT and ERK1/2 proteins, thereby markedly inhibiting migration and invasion of PCa cells (16). Similarly, Ramteke et al. extracted exosomes from LNCaP and PC3 cells exposed to hypoxic (1% O2) and normoxic (21% O2) media and found that CD63, CD81, HSP90, HSP70, Annexin II were expressed at higher levels in the hypoxic environment and that hypoxia enhanced the invasiveness and motility of LNCaP and PC3 cells as confirmed by cell invasion assays. Further research found that this may be related to the above-mentioned proteins promoting the formation of pre-metastatic niche in cancer cells and inducing stem cell proliferation and epithelial–mesenchymal transition (EMT) transformation (38). Furthermore, an exosomal protein study by Jinlu et al. found that the exosomal protein PKM2 secreted by C4-2B cells can be transported to bone marrow stromal cells (BMSC) *via* exosomes and upregulate CXCL12 production in BMSC in a HIF-1α-dependent manner to promote bone metastasis of PCa (45).

**Table 1:** Exosomal protein derived from prostate cancer cell line.

protein	Prostate cancer source	Role in tumors	references
PDCD6IP, FASN, XPO1, ENO1	PNT2C2, RWPE-1, PC346C, and VCaP	Inhibition of apoptosis Involved in lipid metabolism and oncogenic signaling pathways	(6, 12–15)
ITGA3, ITGB1	LNCaP and PC3	Activate oncogenic signaling pathway.	(2, 16, 17)
p-glycoprotein	docetaxel-resistant PC3、PC3	Chemotherapy resistance	(2, 18)
Ets-1	PC3 and DU145	Enhance osteoblast differentiation	(2, 19)
Integrin beta4, vinculin	taxane-resistant PC3	Interacts with proteins to promote tumor metastasis	(20, 21)
ANXA2, CLSTN1, FLNC, FOLH1, GDF15	PC3, DU145, VCaP, LNCaP, C4-2, and RWPE-1	Involved in fat metabolism, cell proliferation, migration and drug resistance, remodeling of cytoskeleton, Angiogenesis, oncogenic signaling pathways	(22–26)
CD9, CD82	LNCaP and PC3	Inhibit the movement of tumor cells, chemoresistance	(27, 28)
CML28	DU145, LNCaP	Activate immunity	(28, 29)
Integrin alphavbeta6	PC3, DU145, C4-2B, RWPE-1	Activating MMP2 promotes the autonomous osteolysis process of cells	(30)
Trop-2	PC3	Activate the metastasis signaling pathway FAK	(31)
CD61, CD81, HSP90, HSP70, Annexin II	PC3, LNCaP	cellular activation、cell motility、tumor cell metastasis、Metabolic reprogramming, mediating immune microenvironment, tumor resistance	(32–38)
TGF-beta	PC stem cells	Proliferation, apoptosis, differentiation, epithelial -mesenchymal transition (EMT) and migration	(38, 39)
Rab1a, Rab1b, Rab11a	C4-2B	Tumor reprogramming of patient-derived adipose stem cells promotes tumor proliferation	(40)
CD276	DU145, 22Rv1, and LNCaP	Acts as a T cell inhibitor to promote tumor proliferation and invasion	(41)
δ-catenin	PC3	Interacts with E-cadherin to inhibit tumor migration	(42, 43)
LDHA	VCaP, LNCaP, C4-2B	Cell metabolism	(22, 44)
CLU, FN1, KRT8, LAMA5, NPM1, PRDX1, TFRC	DU145, PC3 cells	Regulate cell death and intercellular signaling	(22)
PKM2	LNCaP, DU145, and PC3	Promote the expression of CXCL12 in stromal cells	(45)
Claudin 3	DU145	Increase cell motility and survival by activating MMP -2/Suppression of EMT	(46, 47)
MDR-1、MDR-3、Endophilin-A2 、PABP4、PACSIN2	U145 Tax-Res	Chemotherapy resistance	(48)
Caveolin-1	PC3	suppresses tumor formation through the inhibition of the unfolded protein response	(49)
CD147 、CD44	U145 Tax-Res	Activation of PI3K and MAPK pathways mediate tumor me -tastasis and chemotherapy resistance	(50, 51)
ACTN4	DU145	Promote the movement and proliferation of tumor cells	(52, 53)

### PCaDEPr in Plasma

Studies evaluating the clinical value of exosomal proteins in PCa have confirmed that plasma-derived exosomal proteins play a key role in tumor survival and metastasis, among others (**Table 2**). Notably, PCa patients exhibit significantly higher levels of proteins involved in substance metabolism (P-gp), bioactive enzymes (NEU3, C1r), and cell survival (Survivin, PIF1) in their plasma exosomes, relative to cell lines, and have reportedly been associated with a variety of tumor survival and metastasis. Bergelson et al. found that NEU3 is highly expressed in various cancers such as colon cancer and renal cancer, and significantly inhibits the apoptosis of cancer cells. Another study found that NEU3, as an enzyme that specifically hydrolyzes gangliosides, can reduce the ganglioside-mediated immune activation process (54). This result suggests that NEU3 may act as an immunosuppressant in tumors. Kishi et al. detected the expression of Survivin in the tissues of 82 PCa patients and found that its expression was positively correlated with the pathological stage, Gleason score (ranges from 1-5 and describes how much the cancer from a biopsy looks like healthy tissue (lower score) or abnormal tissue (higher score)) and cell proliferation activity of PCa, and could inhibit cell apoptosis (58). Additional research evidences have shown that plasma exosomal proteins may also have bidirectional effects on tumors. For example, P-gp in exosomes was reportedly elevated in doxorubicin-resistant PCa (18), while another study showed that it enhanced the anti-cancer ability of anti-cancer cytokines, such as CD4^+^ T cells, in ovarian cancer (84). Conversely P-gp was also found to activate expression of pro-tumor progressive M2 type macrophages (85). Collectively, these findings suggest that plasma exosomeal protein P-gp may be a potential therapeutic target for tumors.

**Table 2:** Exosomal proteins in the blood of prostate cancer patients.

protein	Role in tumors	references
NEU3	Immunosuppressive	(2, 54, 55)
p-glycoprotein	Chemotherapy resistance	(18)
CYP17A1、CYP17	Activate AR	(56)
HSP72	Activate immunity	(57)
Survivin	Inhibit apoptosis	(58, 59)
CML28	Promote cell proliferation	(28, 29)
αvβ3 integrin	Participate in cell migration	(60)
Claudin 3	Tumor metastasis	(61)
DNA Helicase Homolog PIF1	suppresses Apoptosis	(62)
Four and a Half LIM Domain 3	Protein interaction	(63)
Glutathione S Transferase Omega 2	Participate in cell metabolism	(64)
Maternal Embryonic Leucine Zipper Kinase	Chemotherapy resistance	(65)
Iroquois Homeobox Protein 5	Promote cell proliferation	(66)
Leucine Rich Zipper Containing 4	Enhance cell migration	(67)
Minichromosome Maintenance complex Component 5	Enhance cell migration	(68)
Mitochondrial Tumor Suppressor 1 Isoform 4	Increase cell proliferation and invasion	(69)
Nasopharyngeal epithelium Specific Protein	Interfering oncogenes	(70)
Ubiquitin-like with PHD and ring finger domains	Interfering oncogenes	(71)
Trinucleotide repeat containing 6B Isoform 3	Promote cell proliferation	(72)
Apolipoprotein E (isoform E2)	Protein interaction	(73, 74)
C3a anaphylatoxin des Arginine	Inhibit T cell toxicity	(74, 75)
Complement C1q subcomponent	Promote angiogenesis、Promote immune suppression	(74, 76)
Complement C1r subcomponent	Inhibit apoptosis、Promote angiogenesis	(74, 77)
D-dimer	Promote angiogenesis	(74, 78)
Fibrinogen	Changes in the tumor microenvironment	(74, 79)
Fibrinogen gamma chain	Interaction with FGF-2 promotes cancer growth	(74, 79)
Fibronectin	Interacts with proteins to promote tumor progression or inhibit tumor survival	(74, 80)
Properdin	Activate the complement system to inhibit tumor survival	(74, 81)
von Willebrand factor	mediate multiple cell–cell interactions	(74, 82)
PTEN	Tumor suppression	(83)
ACTN4	Promote the movement and proliferation of tumor cells	(52, 53)

### PCaDEPr in Urine

Numerous studies have shown that urinary exosomal proteins from PCa patients play a non-negligible role in tumors (**Table 3**). The urine exosomes are more abundant in substance synthesis (Sepiapterin), signaling (Ras GTPase, Flot-2), tight junction (Claudin-3, δ-catenin) and other proteins compared to the plasma exosomes. Wu et al. compared SMMC-7721 containing epiapterin reductase (SPR) with SMMC-7721(human hepatocarcinoma cells) containing this mutant and concluded that SPR might be a tumor promoter in HCC (hepatocellular carcinoma). Results from further cellular experiments, as well as analysis of a nude mouse xenograft model, revealed that SPR depletion inhibited HCC cell proliferation and promoted apoptosis, affirming that SPR may regulate hepatocellular carcinoma progression *via* the FoxO3a/Bim pathway (a transcriptional target in apoptosis regulation *in vivo* and *in vitro*) *(* 99). On the other hand, Hazarika et al. performed immunohistochemical staining of Flot-2 and found significantly higher intensities in metastatic melanoma from lymph nodes or visceral sites relative to those in nevi and primary melanoma and their results indicated that overexpression of Flot-2 promoted tumor cell proliferation and vascular regeneration (124). In addition, Flot-2 reportedly plays a role in promoting tumor metastasis, such as and has been shown to induce metastasis in nasopharyngeal carcinoma by activating the NF-κB and PI3K/Akt3 signaling pathways (125). This factor has also been shown to regulate the cell cycle and induce EMT, thereby promoting growth and metastasis of HCC (126). Lin et al. found that knocking down the expression of Claudin 3(CLDN3) resulted in significant changes in the phenotype of ovarian cancer cells, and further studies found that this would significantly downregulate the expression level of E-cadherin and upregulate the expression of N-cadherin. Therefore, CLDN3 may be involved in regulation of the EMT to promote metastasis in ovarian cancer (47). Exploration of the value of urinary exosomal proteins during early diagnosis of PCa is of great importance for subsequent clinical use, owing to the ease of obtaining urine samples. Results from differential protein analysis between healthy men and PCa patients revealed that 246 proteins were differentially expressed, 221 of which were significantly upregulated in exosomes of PCa patients (86). Taken together, these findings suggest that exosomal proteins may have potential as diagnostic and therapeutic markers in PCa.

**Table 3:** Exosomal protein in urine of prostate cancer patients.

protein (86)	Role in tumors	references
PPP2CA	Reverse EMT transformation to inhibit prostate tumor growth and metastasis	(87)
Rab-35	Induced EMT、intracellular signaling、apico-basal polarity、cytokinesis and cell migration Promote the differentiation and proliferation of tumor cells	(88)
S100-A6	S100A6 interacts with annexin 2 promotes cancer cell motility	(89)
P2X purinoceptor 4	Induction of immunosuppression and angiogenesis, Activate anti-tumor response	(90)
Galectin-3	These include inhibition of apoptosis, promotion of cell growth, and regulation of TCR signal transduction, promotes angiogenesis	(91–94)
flotillin-2	Molecules involved in signal transduction, adhesion, and extracellular matrix remodeling	(95)
Calmodulin	The interaction of CaM and AR promotes the proliferation of LNCaP cells	(96)
3-hydroxybutyrate dehydrogenase type 2	Induce apoptosis	(97)
Thioredoxin domain-containing protein 17	Induces autophagy to promote chemotherapy resistance	(98)
Sepiapterin reductase	Regulate FoxO3a、Bim signal to promote tumor progression、Induce ROS-mediated apoptosis and inhibit tumor cell proliferation	(99, 100)
Melanophilin	Accelerate EMT to promote tumor metastasis	(101)
MFSD12	Promote G1 phase	(102)
LIMP-2(Lysosome membrane protein 2)	Transport lysosome	(103)
Glucosamine-6-phosphate isomerase 1	Promote metabolism and inhibit apoptosis	(104)
GDP-mannose 4.6 dehydratase	Regulate TRAIL-induced apoptosis and increase NK cell-mediated tumor surveillance	(105)
Claudin-3	Increase cell motility and survival by activating MMP-2/Suppression of EMT	(46, 47)
Claudin-2	Epithelial-mesenchymal transition (EMT), tumor initiation, and chemotherapy resistance	(61)
Claudin-10	Transforming growth factor-β (TGF-β)- or WNT/β-catenin-induced EMT affects the progress of OC	(106)
Tetraspanin-6	Regulate EGFR-dependent signaling	(107)
Proton myo-inositol cotransporter	Regulate Hif-1α to promote tumor cell hypoxia	(108)
ADP-ribosylation factor-like protein 8B	Lysosomal transport	(109)
Synaptotagmin-like protein 4	Chemotherapy resistance	(110)
Protein S100-P	Chemotherapy resistance	(86, 111)
Protein DJ-1	Inhibit PTEN tumor suppressor	(112)
Metalloreductase STEAP4	Involved in the metabolism of cell iron and copper	(113)
ATP6V0C	Enhance the function of V-ATPase to promote the migration and invasion of cancer cells	(114)
Ras-related protein Rab-7a	Prevent HGF-induced lysosomal trafficking, cathepsin B secretion and cell invasion	(115)
Ras-related protein Rab-3D	Induces cytoskeleton remodeling, enhances cancer cell movement, induces EMT, regulates Hsp90α secretion and promotes tumor cell invasion	(116)
Ras-related protein Rab-3B	Inhibit apoptosis and maintain cancer cell survival	(117)
Ras-related protein Rab-2A	Activate Erk signal to promote breast cancer stem cells and tumorigenesis	(118)
Plastin-2	Regulate integrin-mediated tumor cell adhesion	(119)
Ragulator complex protein LAMTOR1	Affect lysosomal localization	(120)
ADIRF	Induce PPARG expression to promote adipocyte differentiation	(121)
PSA, PSMA	Related to angiogenesis	(2, 122)
δ-catenin	Interacts with E-cadherin to inhibit tumor migration	(42, 43)
ITGA3, ITGB1	Activate oncogenic signaling pathway	(16)
Transmembrane Protein 256	Induce tumor formation	(123)

### PCaDEPr in Tissues

Apart from plasma and urine exosomal proteins from PCa patients, exosomal proteins from PCa tissues have also been extensively studied (**Table 4**). For example, results from mass spectrometry analysis revealed that PCa tissue exosomal protein types are mainly involved in vesicle transport and composition (Annexin A5, Annexin A3), biotransformation enzymes (such as Glutathione synthetase, and D-3-phosphoglycerate dehydrogenase), cytoskeletal molecules (Syntenin-1) and other related proteins. Moreover, previous studies have confirmed that these proteins play a role in tumor initiation and progression. Tang et al. demonstrated that Annexin A5 could activate the PI3K/Akt/mTOR signaling pathway to regulate the EMT process and matrix metalloproteinase (MMP) expression thereby significantly promoting proliferation, migration and invasion of renal cancer cells both *in vitro* and *in vivo (* 145). Kennedy et al. reported that Glutathione plays a role as an intracellular antioxidant in cancer, a where it regulates reactive oxygen species (ROS)-mediated signaling pathways, including NF-kB and MAPK/ERK, to maintain tumor survival and induce tumorigenesis (128). ROS, which are closely related to Glutathione, were found to regulate Cav-1 expression in human lung cancer H460 cells, thereby modulating their migration and invasion. However, different ROS exert different effects in tumors. Superoxide anion and hydrogen peroxide were found to significantly downregulate Cav-1 expression and inhibit both cell migration and invasion, while hydroxyl radicals reportedly upregulated Cav-1 expression and also promoted cell migration and invasion (163). With regards to chemoresistance, Iwamoto et al. reported that Syntenin-1 was upregulated in rectal cancer (CRC) tumor tissues, while its downregulation mediated a significant downregulation of prostaglandin E2 receptor (PTGER2). On the other hand, silencing PTGER2 decreased chemoresistance of cancer stem cells to oxaliplatin. Taken together, these results indicated that Syntenin-1 may promote chemoresistance in cancer cells (147).

**Table 4:** Exosomal proteins in prostate cancer tissue.

protein (127)	Role in tumors	references
Glutathione synthetase	Inhibit oxidative stress, tumor progression and chemotherapy resistance	(128)
D-3-phosphoglycerate dehydrogenase	Up-regulation of cancer-promoting genes, regulation of metabolism, chemotherapy resistance	(129)
Cytosol aminopeptidase	Affects MHC class I mediated antigen presentation	(130)
Alpha-enolase	Protein-protein interactions that regulate glycolysis, activation of signaling pathways, and resistance to chemotherapy	(131)
Keratin, type I cytoskeletal 10	Inhibit cell cycle progression	(132)
Actin, cytoplasmic 1	Causes cytoskeletal changes to promote tumor progression	(133)
Isocitrate dehydrogenase 1 (NADP+), soluble	Control lipid metabolism and inhibit apoptosis	(134)
Alcohol dehydrogenase [NADP+]	Activate the carcinogenic effects of acetaldehyde	(135)
Sorbitol dehydrogenase	Inhibit cell hypoxia	(136)
F-Actin-capping protein subunit alpha-1	Remodeling the cytoskeleton inhibits EMT, thereby inhibiting cancer migration and invasion	(137)
N(G), N(G)-Dimethylarginine dimethylaminohydrolase 1	Inhibit angiogenesis	(138)
Annexin A1	Induces apoptosis, activates immunity, mediates cancer pathways, and protein interactions	(139–143)
14-3-3 Protein sigma	Induces cell cycle arrest and apoptosis of cancer cells, affects transcription factors and cell signal transduction in cancer cells, and resists oxidative stress	(144)
Annexin A5	Annexin A5 can activate the PI3K/Akt/mTOR signaling pathway to promote epithelial-mesenchymal transition (EMT) and the expression of MMP2 and MMP9	(145)
Annexin A3	Participate in cell signal transduction and promote tumor development	(146)
Syntenin-1	Regulating PTGER2 expression enhances CSC amplification, oxaliplatin chemoresistance and migration	(147)
Heat-shock protein beta-1	Inhibit cell apoptosis in various malignant tumors, up-regulate the expression of MMP-9, promote the invasion of breast cancer cells, and increase VEGF) to induce angiogenesis	(148–151)
Peroxiredoxin-6	Regulate the expression of uPAR, Ets-1, MMP-9, RhoC and TIMP-2 to increase the invasion and metastasis of breast cancer	(152)
Triosephosphate isomerase	Regulate glycolysis and metabolism, as an oncogene	(153)
Phosphatidylethanolamine-binding protein 1	Inhibit most of the kinase functions in the signal cascade, metastasis inhibitors, participate in cell proliferation, inhibit metastasis, and promote apoptosis	(154)
Semenogelin-1	Activate androgen receptor	(155)
Superoxide dismutase [Cu-Zn]	Inhibit the oxidative stress response of cells	(156)
Ubiquitin-conjugating enzyme E2 N	Involved in DNA repair, cell cycle progression, cell apoptosis and carcinogenic signals	(157)
Prolactin-inducible protein	Enhance anti-tumor immunity and promote tumor metastasis	(158)
Protein S100-A9	Regulate tumor immune microenvironment	(159)
Histidine triad nucleotide-binding protein 1	Inhibition of oncogene transcriptional control pathways	(160)
Acyl-CoA-binding protein	Maintain fatty acid oxidation to induce tumorigenesis	(161)
Protein S100-A11	Regulate cell cycle, promote cell proliferatio n, migration, invasion and EMT, activate Wnt, β-catenin signaling pathway to induce cancer	(162)

## PCaDEPr in Tumor Cell Initiation and Proliferation

The basic understanding of tumorigenesis is uncontrolled cell proliferation or uncontrolled apoptosis. In addition to changes in tumor cells, changes also occur in the tumor microenvironment, including variations in structure and function of stromal cells such as fibroblasts, lymphocytes, epithelial cells, and matrix molecules, like growth factors and cytokines. Previous studies have shown that PCaDEPr can induce tumor cell initiation and proliferation processes by regulating metabolic, apoptotic, and TME pathways.

### Inhibition of Apoptosis Induces Cell Tumorigenesis

Apoptosis is an orderly and coordinated process of cell death that occurs under physiological and pathological conditions. In cancer, dying cells do not receive apoptotic signals, due to an imbalance between cell division and cell death, a phenomenon that causes normal cells to tumorize. Apoptosis can induce development of cancer cells through intrinsic and extrinsic pathways, which involve action of many proteins that regulate apoptosis and these proteins are also present in prostate cancer-derived exosomes. For example, Sepiapterin reductase (SPR), an important regulator of tetrahydrobiopterin (BH4) biosynthesis, has been shown to be a promoter of various tumors. Zhang et al. found that ROS-mediated apoptosis could be induced by knocking down SPR expression to inhibit progression of breast cancer cells (100). Similarly, Basu et al. found a strong association between S100 Calcium Binding Protein P (S100P) expression and prostate tumorigenesis, with S100P expression mediating basal apoptosis and impeding camptothecin-induced apoptosis. Moreover, silencing of S100P significantly inhibited growth of 22Rv1 cells, while overexpressing S100P in PC3 cells resulted in increased proliferation of tumor cells (164). In addition, other exosomal proteins interact with apoptosis-related proteins to induce tumorigenesis. Ingo et al. found that ornithine decarboxylase (ODC) and Sepiapterin reductase (SPR) proteins interacted to elevate ODC activity, thereby inhibiting apoptosis and inducing neuroblastoma cell genesis (165). Exosomal protein phosphatase and tensin homolog (PTEN) was shown to negatively regulate expression of the cyclin-dependent kinase (CDK) inhibitor p27 (KIP1), thereby inhibiting apoptosis (166). Previous studies have shown that S100 calcium-binding protein A6 (S100A6) interacts with p53 to affect oligomerization and activity of p53, thereby reducing its ability to promote apoptosis (167, 168). Moreover, tumor cells can also induce cancer by secreting exosomes to eliminate proteins that initiate apoptosis. Diederick et al. showed that cancer cells can remove PDCD6IP, a protein involved in programmed cell death, by exosome secretion to inhibit apoptosis, explained by high PDCD6IP abundance in PCa-derived exosomes and low abundance in autologous tumor cells this possibility (6). Collectively, these studies indicate that exosomal proteins can induce tumorigenesis and proliferation by inhibiting the apoptotic process both directly and indirectly.

### Involvement in Substance Metabolism and Oxidative Stress

Alteration of pathways regulating cellular substance metabolism is more common in cancer, compared to normal tissue cells. In fact, alterations in normal cellular substance metabolism have been implicated in convergence of cells to a tumor state. Previous studies have shown that alterations in tumor metabolism include glycolysis, lipid hydrolysis, increased nutrient utilization, and increased production of biosynthetic intermediates required for cell growth and proliferation. Liu et al. found that Fatty acid synthase (FASN) protein was upregulated in exosomes derived from Vertebral-Cancer of the Prostate (VCaP) cells. Additional studies have demonstrated that FASN catalyzes formation of long-chain fatty acids from acetyl coenzyme A, malonyl coenzyme A and NADPH, to promote proliferation of VCaP cells, and that inhibition of FASN effectively and selectively kills cancer cells (166). Qin et al. demonstrated that ADP-ribosylation factor-like 8b (Arl8b) Arl8b depletion reduced the ability of PCa cells to establish subcutaneous xenografts in mice. Under a low nutrient environment, Arl8b maintained efficient metabolism in PCa cells thereby allowing them maintain their excessive proliferative capacity by promoting lipid hydrolysis. Metabolic defects in the proliferation of cells with low Arl8b expression inhibit tumor growth initiation *in vivo*. The phenomenon may be attributed to the fact that Arl8b depletion impairs intracellular neutral lipid hydrolysis, thereby shifting the metabolic profile to an abnormal lipogenic phenotype, which subsequently impairs glucose utilization and limits the propensity for cytokinesis (169). In addition, Webber et al. demonstrated that the exosomal protein TGFβ1 secreted by cancer-associated fibroblasts enhanced proliferation of PCa cells under both hypoxic and low nutrient environments by inhibiting mitochondrial oxidative phosphorylation and elevating anaerobic glycolysis (170).

Oxidative stress, a series of adaptive responses caused by an imbalance between ROS and the body’s antioxidant system, plays a key role in cancer development and progression. Previous studies have shown that by interfering with the normal redox state of cells, oxidative stress causes generation of peroxides and free radicals that subsequently damage cellular proteins, lipids and DNA, thereby causing tumor development. For example, ROS can either initiate or stimulate tumorigenesis and support the transformation and proliferation of cancer cells. Over-proliferation of tumor cells is often accompanied by high ROS production, and thrives under such oxidative load conditions. At the same time, tumor cells can optimize the ROS-driven cell proliferation process by increasing their antioxidant capacity, to avoid the ROS threshold that triggers senescence, apoptosis and iron-induced cell death (171). Previous studies have shown that some proteins in exosomes can influence ROS expression during oxidative stress in cells, thereby affecting the tumor initiation process. For example, Qin et al. reported that ectopic expression of six transmembrane epithelial antigen of prostate 4 (STEAP4) in PCa cells significantly increased tumor cell proliferation and colony formation, suggesting that STEAP4 may be playing a role in tumor growth. This phenomenon may be explained by the fact that high STEAP4 expression not only downregulates IRS-1, PI3K and AKT phosphorylation but also impairs insulin-mediated GLUT4 translocation, thereby resulting in ROS-associated mitochondrial dysfunction (169). In another study, silencing of STEAP4 significantly inhibited growth of mouse PCa xenografts in a mouse model. Furthermore, STEAP4 expression was found to mediate elevation of ROS levels probably by increasing levels of ferrous iron in cells after using it as a redox intermediate (electron donor) to generate free radicals. In addition, STEAP4 expression reportedly depleted production of NADPH (172), an inhibitor of ROS production, thereby resulting elevated ROS production. Notably, persistently high ROS levels were found to promote cancer development, owing to is oncogenic nature (113).

### Activation of TME Regulatory and Tumorigenic Signaling Pathways

The tumor microenvironment refers to the surrounding microenvironment where tumor cells exist, including surrounding blood vessels, immune cells, fibroblasts, bone marrow-derived inflammatory cells, adipose stem cells and various signaling molecules and the extracellular matrix (ECM). During early stages of cancer development, tumor cells appropriately regulate the microenvironment. For example, various microenvironmental changes, such as adjustment of the ECM, immune response, stromal stem cell transformation and induction of angiogenesis, can be triggered during tumor initiation (173). Recent studies have shown that PCaDEPr may promote production of tumor cells through this pathway. Zakaria et al. found that the PCa cell microenvironment disrupts adipose-derived stem cells in PCa patients to induce tumor transformation, but unlike normal stem cells, the use of PCa cell-conditioned medium effectively triggers conversion of adipose-derived stem cells into prostate-like tumor lesions *in vivo*. Furthermore, exosomal proteins, namely Rab1a, Rab1b, and Rab11a, in PCa were found to recapitulate the formation of prostate tumorigenic mimics generated by adipose-derived stem cells triggered by PCa cell conditioned medium. In fact, the use of PCa cell-derived conditioned medium (CM) or exosomes was found to effectively trigger adipose stem cells to undergo genetic instability, mesenchymal-to-epithelial transformation (MET), and oncogenic transformation, thereby inducing PCa *in vivo*. This may be explained by the fact that exosomes deliver oncogenic factors, such as Rab proteins (Rab1a, Rab1b, and Rab11a) translocated to pASCs to inhibit large tumor suppressor kinase 2 (LATS2) and programmed downregulation of cell death protein 4 (PDCD4), thereby promoting tumor growth (40). In the tumor vascular microenvironment, Dominique et al. showed that HSP27 interacted with CD283, thereby inducing NF-κB activation, which subsequently led to vascular endothelial growth factor (VEGF)-mediated angiogenesis in the tumor microenvironment (174).

In addition, PCaDEPrs have also been shown to induce tumorigenesis by modulating alterations in tumorigenic signaling pathways. For example, flotillins that are also present in tumor-derived exosomes. Jang et al. showed that palmitoylation of Flot-1 could regulate proliferation of PCa cells by activating the IGF-1R signaling pathway. Moreover, palmitoylation (S-palmitoylation) modification of Flot-1 was found to regulate intracellular signaling proteins p53, STAT1 (signal transducer and activator of transcription 1) and IκBα (nuclear factor of kappa light polypeptide gene enhancer in B-cells inhibitor, alpha), thereby inducing oncogenic effects (175). Furthermore, Takahashi-Niki et al. found that Parkinson disease protein 7 (DJ-1) binds to Topors/p53BP3 (176), both *in vitro* and *in vivo*, thereby releasing the monoglycosylated form of p53 and helping to restore the transcriptional activity of p53. Recent research evidence showed that DJ-1 directly binds to Sirtuin1 (Sirt1), to stimulate Sirt1 deacetylase activity. Furthermore, DJ-1 downregulated the transcriptional activity of sirt1-suppressed sirt1 target p53 (177). Taken together, these results indicated that p53 is closely associated with DJ-1, suggesting presence of a finely regulated circuit between both proteins during tumorigenesis and apoptosis. Previous studies have also shown that major facilitator superfamily domain containing 12 (MFSD12), which is highly expressed in melanoma, induces proliferation of melanoma cells *via* the PI3K- AKT signaling pathway (102).

### Interference With the Cell Cycle

Continued unregulated growth of cancer cells is a fundamental abnormality during cancer development and progression. In fact, the first step in the process, tumor initiation, is believed to be the basis for initiation of abnormal proliferation of individual cells. Subsequent cell proliferation causes growth of clonally derived tumor cell populations. Numerous studies have identified a number of proteins that regulate proliferation of the cell cycle, to subsequently trigger tumorigenesis. For example, one study showed that estrogen stimulates the proliferative cycle of endometrial cells, while exposure to excess estrogen significantly increases the risk of endometrial cancer in women (178). PCaDE also contains similar proteins that interfere with cell cycle processes, to promote tumor cell development. For instance, Bosch et al. demonstrated that Ca^2+^ and calmodulin (CaM) play a key role in proliferation and viability of a variety of cells, including PCa. This phenomenon may be attributed to the fact that CaM interacts with various proteins that regulate the cell cycle, including p21Cip1, D1-Cdk4 and CaM kinase II, to control their activities and nuclear localization, thereby influencing proliferation of tumor cells (179). Moreover, cell cycle protein A in LNCaP cellular extracts was found to directly or indirectly bind to CaM, indicating that its expression is sensitive to the inhibitory effect of the anti-CaM drug W-7. Notably, this indicates that CaM regulates expression of cell cycle protein A in PCa cells to induce over-proliferation of LNCaP cells (96). In addition, previous studies have shown that expression of MFSD12, a novel suppressor gene in lung cancer, and its protein, can control cell cycle distribution, matrix attachment and cell motility, thereby regulating tumor growth and development. MFSD12 was significantly upregulated in melanoma tissues, with interreference in its expression in A2058 and M14 melanoma cells found to significantly suppress tumor cell proliferation. Results from flow cytometry analysis confirmed that silencing MFSD12 expression mediated increase and decrease in the proportion of cells in the G1and S phases, respectively, suggesting that MFSD12-induced proliferation is associated with promotion of the G1 phase (102).

## PCaDEPr in Cancer Survival and progression

Evasion of death is imperative to cancer cells’ persistence and their subsequent progression. Several tumor survival proteins are present in the tumor survival microenvironment, where they play a key role in regulatory processes including apoptosis (180), metabolism (181), immune escape, nutrient transport, hypoxic environment and drug resistance, that promote tumor cell survival. Proteins related to apoptosis and also present in PCa exosomes, such as Bcl-2, inhibitor of apoptosis (IAP) and heat shock protein (HSP) and proteins related to cell metabolism, such as glucose transporter 1 (GLUT1) and Ras, etc. Considered a family of survivin proteins of tumor cells (180). Abnormal expression of these proteins is associated with a series of biological regulatory processes that promote cancer cell survival, proliferation, and treatment resistance (181). In addition, cancer cells employ progression as a means for tumors to maintain survival, thus tumor survival is closely associated with progression. Tumor progression is characterized by rapid changes in the tumor phenotype, a phenomenon that has made tumors to become more aggressive. Exosomal proteins are thought to play various roles in progression of various tumor types, including remodeling of the tumor microenvironment, promoting epithelial mesenchymal transition (EMT), angiogenesis induction, promoting migration, invasion and immune escape of cancer cells, as well as regulating the corresponding signaling pathways (182).

### Remodeling the Tumor Microenvironment

Immune escape is an important aspect in tumor survival, as tumor cells can only proliferate, migrate and invade tissues if they escape killing by immune cells, such as phagocytes, T cells, and NK cells. The exosomal proteins, which are secreted by cancer cells support immune escape to promote tumor cell survival. For instance, exosomal proteins support immune cell migration (such as neutrophils, macrophages and regulatory T cells) to secondary sites, suppress immune responses to tumors by inhibiting the efficacies of antigen-presenting cells, such as dendritic cells. They can also impair immune functions of T and NK cells by activating apoptosis (183, 184). Moriwaki et al. demonstrates that tumor cells lacking GDP-mannose-4,6-dehydratase (GMDS) can evade NK cell-mediated tumor immune surveillance by acquiring resistance to tumor necrosis factor-related apoptosis-inducing ligand (TRAIL)-induced apoptosis (105). Aled et al. found that TGF-β-positive exosomes downregulated natural-killer group 2, member D(NKG2D) expressions in NK and CD8+ T cells, which in turn impaired immune effector functions (185). TGF-β-rich exosome inhibits lymphocyte responses to IL-2, thereby altering the tumor microenvironment to promote immune escape functions of tumor cells (186). In addition, documented those expressions of some purinergic receptors directly or indirectly inhibit T cells and NK cells effects, thereby suppressing immune responses to primary tumors. For instance, oncogenic exosomes with elevated CD39 and CD73 levels can promote adenosine production, thereby enhancing regulatory T cell and myeloid cell proliferation to suppress immune functions (187). Interestingly, exsomeal proteins have also been shown to promote tumor progression by activating tumor-associated immune cells. Wang et al. found that LAMP 2a contributes to tumor progression by degrading PRDX1 (peroxiredoxin 1) and CRTC1 (CREB-regulated transcriptional coactivator 1), which enhances tumor-associated macrophage activation (188). These studies confirm that cancer-derived exosomal proteins can mediate the escape of tumor cells from immune surveillance to promote their survivability.

In addition to regulation of immune microenvironments, exosomal proteins are involved in construction of other tumor microenvironments to maintain tumor survival. For example, tumor cell over proliferation leads to the development of hypoxic environments, therefore, the ability to regulate tumor cell tolerance to hypoxic environments is necessary for tumor cell survival, and some proteins in exosome can play this function. Hypoxia-inducible factor-1α (HIF-1α), a master transcription factor, is stable under hypoxic conditions. It regulates the expressions of several target genes and enhances the adaptability of tumor cells to hypoxia (189). Zhong et al. found that under hypoxic conditions, DJ-1 is involved in regulation of HIF-1α transcriptional activities, promoting PCa adaptation to hypoxic environments (190). Inflammatory microenvironments can also affect tumor survival and progression. For instance, DJ-1 is involved in creation of inflammatory tumor microenvironments. In their study, Chien et al. found elevated levels of IL-1β in cultured macrophages from DJ-1 DJ-1 Knockdown mice and DJ-1 knockdown mice (191). It was also confirmed that the inflammatory microenvironment generated by DJ-1 dysregulation sustained melanoma survival at the point of lung metastasis. Another study found that adipose-derived stem cells (ADSCs) induced by exosomal proteins exhibited typical characteristics of tumor-associated myofibroblasts, and could induce the phenotype and function of myofibroblasts in ADSCs by activating intracellular signaling pathways. Increased expression of smooth muscle actin (α-SMA) and tumor-promoting factors such as the stromal cell-derived factor 1 (SDF-1) and TGF-β. These outcomes are associated with increased expressions of TGF-β receptors I and II in exosomes (189). Therefore, exosomal proteins contribute to the generation of tumor-associated myofibroblasts in the tumor stroma to construct an extracellular matrix environment suitable for tumor survival.

### Hormone Receptor Regulation and Metabolic Reprogramming

Although the emergence of castration-resistant PCa poses difficulties for androgen deprivation therapy (ADT), androgen depletion and hormonal regulation have been the mainstay of advanced disease treatment since the landmark discovery of Huggins and Hodges (192). The PCaDEPr that are associated with hormone receptor regulation, including adhesion spot protein (VCL), play an important role in cancer progression. Kawakami et al. reported that the VCL, through which integrins associate with the actin cytoskeleton, promotes paclitaxel resistance-associated PCa invasion. They found that VCL levels were highest in CRPC, negative or very low in BPH and non-CRPC, and confirmed that VCL overexpressions promotes PCa progression by altering androgen receptor (AR) levels (21). Iwamoto et al. found that Syntenin-1 levels are positively correlated with prostaglandin E2 receptor (PTGER2) levels and promotes rectal cancer cell progression (147). In addition to regulation of hormone receptors, these proteins mediate hormone levels, thereby activating hormone receptors. Abiraterone acetate (CYP17A1), an integrase involved in adrenal steroid conversion and *de novo* synthesis of androgens, is involved in CRPC production. Attard et al. used an inhibitor of CYP17A1 synthesis to treat 21 desmoresistant PCa patients. They reported a decrease in serum androstenedione, dehydroepiandrosterone (DHEA) and testosterone levels *in vivo*, and in CRPC patients. The antifungal drug, ketoconazole, is similar to CYP17A1 inhibitors and suppresses testosterone synthesis (193). Therefore, CYP17A1 promotes androgen expressions, which have a significant role in AR activation. Therefore, PCaDEPr mediated regulation of hormone receptors may be one of the pathways in cancer progression.

Metabolic processes are crucial for cell survival, especially tumor cells. The exosomal proteins have the ability to regulate substance metabolism-related proteins to induce metabolic reprogramming and provide energy as well as biosynthetic pathways to tumor cells. For example, glucose transporter protein 1 (GLUT1) regulates cellular glucose uptake and responds to suppressed intracytoplasmic glucose levels. Cheng et al. found that Rab25(member RAS oncogene family) regulates GLUT1 transport to cell surfaces to enhance glucose uptake and ultimately increases glycogen reserves as well as ATP levels in ovarian cancer cells (194). Even though dysregulated glucose metabolism is important for metabolic reprogramming in tumor cells, metabolic reprogramming in tumor cells also involves lipid storage and mobilization. Walther et al. found that Rab GTPases regulates GLUT (glucose transporter protein) transport and lipid droplet (LD) formation during glucose and lipid metabolism in cancer cells. Lipid droplets (LD) have a role in intracellular lipid storage and maintenance of intracellular levels of free lipids and energy homeostasis (195). Wu et al. reported that Rab8a regulates lipid droplet fusion and cancer cell growth in hepatocellular carcinoma (196), thereby maintaining hepatocellular carcinoma cell survival.

### Regulation of Lysosomal Functions and Distribution

Lysosomes are important components of the endosomal system. They are involved in various biological processes, including macromolecular degradation, antigen presentation, intracellular pathogen destruction, plasma membrane repair, exosomesrelease, cell adhesion/migration, and apoptosis. Functional states and spatial distributions of lysosomes are closely associated with cancer cell proliferation, energy metabolism, invasion and metastasis, as well as immune escape. Invasiveness of radiation-surviving cancer cells is associated with altered lysosomal exocytosis induced by activation of Arl8b present in prostate cancer-derived exosomes. Ping-Hsiu Wu et al. found that after radiation, Arl8b, a small GTPase that regulates lysosomal transport, increased its binding to its effector-SifA and kinesin-interacting protein (SKIP) through the regulation of the BORC (Biogenesis of lysosome-related organelles complex) subunit. Knockdown of Arl8b or the BORC subunit suppressed lysosomal cytokinesis and invasiveness of radiation-surviving breast cancer tumor cells. *In vivo*, suppression of Arl8b levels inhibited radiation-induced invasive tumor growth and distant metastasis (109). Moreover, Arl8b is also a key regulator of lysosomal localization (197). The active form of Arl8b is mainly located in the lysosome, where it regulates lysosomal transport to the cell periphery (198). The cis-transport of lysosomes from the center of microtubule tissues to the cell periphery is regulated by the BORC/Arl8b/SKIP complex (199). Therefore, Arl8b regulates spatial distribution of lysosomes and protease release through lysosomal localization, leading to elevated tumor cell invasiveness. In addition, as a key protein in lysosomal functions, cathepsin D is widely found in PCa-derived exosomes and is associated with tumor progression. Yong et al. found that cathepsin D levels are positively correlated with colorectal cancer malignancy, and that patients with elevated cathepsin D levels have lower survival rates (200).

### Inhibition of Cancer Cell Apoptosis

Homeostatic balance in an organism is maintained by programmed cell death or apoptosis. In addition to being associated with tumor survival, apoptosis is also closely associated late survival of tumor cells. In cancer patients, tumor cells also undergo their own apoptosis, leading to the death of cancer cells. However, some biomolecules such as proteins present in prostate cancer-derived exosomes inhibit this process to keep tumor cells alive. Hahm et al. reported that induction of lysosomal-associated membrane protein 2A (LAMP2A) expression inhibited the apoptotic abilities of prostate cells, thereby enhancing cancer cell survival. LAMP2A protein knockdown in PC-3 and 22Rv1 cells significantly increased the apoptotic rate in both cells, confirming that LAMP2A is involved in induction and activation of the apoptotic protein (Bcl-2) (201). Ding et al. documented that LAMP2A downregulation significantly increased positive apoptosis-staining of hepatocellular carcinoma cells, while decreasing Ki-67(a staining for lipid membranes) staining, confirming that LAMP2A contributes to cancer persistence by inhibiting apoptosis and promoting cell proliferation (202). Moreover, in primary breast cancer samples, DJ-1 levels were negatively correlated with PTEN immunoreactivity and positively correlated with PKB (Protein kinase B)/Akt hyperphosphorylation. Co-expressions of DJ-1 and PTEN completely rescued the apoptotic processes of PTEN-induced tumor cells (112).

Apoptosis affects tumorigenesis and has an important role in late tumor progression. Therefore, interference with apoptosis can promote cancer progression. *In vitro* and *in vivo*, elevated caveolin-1 expressions in metastatic mice and human PCa cells have been reported, suggesting that inhibition of apoptosis promotes tumor progression. Overexpressions of caveolin-1 in LNCaP) or upregulation of Cav-1 in androgen-insensitive LNCaP clones makes these cells resistant to apoptosis (203). Li et al. found a significant association between elevated glucosamine-6-Phosphate Deaminase 1 (GNPDA1) levels and advanced tumor stage, TNM (the TNM classification of malignant tumors) stage or grade, and the subsequent apoptotic staining analysis revealed that elevated GNPDA1 levels inhibited HCC cell apoptosis (104). Therefore, GNPDA1 promotes hepatocellular carcinoma progression by inhibiting HCC cell apoptosis.

## PCaDEPr in Cancer Transfer

Approximately 90% of human cancer-associated deaths are attributed to metastases (204). One of the hallmarks of malignancy is a high degree of invasiveness and metastatic capacity. During development of most cancer types, sooner or later, the primary tumor mass produces free cells that invade adjacent tissues and migrate to distant sites, where they establish new tumor cell colonies. Presumably, the processes involved in invasion and metastasis are: separation from the primary tumor mass, reorganization/remodeling of the extracellular matrix, cell migration, recognition, movement through endothelial cells and vascular circulation, as well as colonization and proliferation within the ectopic stroma. The key and initial to all these processes is an increased ability of cancer cells to move themselves and escape the control of normal physiological regulation. Various biomolecules, including proteins, are involved in regulation of tumor cell invasion and metastasis. This could be because, proteins can influence the tumor microenvironment, EMT, target microenvironment, vascular regeneration, and metastatic signaling pathways to induce distant tumor cell metastasis.

### Establishment of pre-Metastatic Ecological Niches

Tumor cells require a permissive environment in terms of nutrients, extracellular matrix and immune cells to successfully metastasize to distant organs. Therefore, tumor-adapted metastable environments are particularly important for tumor metastasis, and the process of constructing these microenvironments involve the establishment of pre-metastatic ecological niches. Studies on metabolic networks and seeding mechanisms of cancer cells in specific environments have revealed that some integrin proteins are involved in establishment of these ecological niches. During metastasis, tumor cells must acquire the ability to remodel the extracellular matrix (ECM) to achieve invasion and metastasis. Some exosomal proteins are involved in regulation of this process. Bijnsdorp et al. found that Integrin Subunit Alpha 3 (ITGA3) and Integrin beta-1 (ITGB1) were highly expressed in urinary esosomes of metastatic PCa patients, and that ITGA3 and ITGB1, as well as ITGA3 in exosomes, stimulated non-cancerous epithelial cell migration and invasion. This enables the progression and distant metastasis of cancer cells (205). Moreover, the immunosuppressive microenvironment is important in development of pre-metastatic niches, and some exosomal proteins in PCa are involved in establishment of immunosuppressive microenvironments. Allard et al. found that synergistic actions of two extracellular nucleotidases (CD39 and CD73), constituted the main source of extracellular adenosine in TME and were jointly involved in development of immunosuppressive TME, such as through tumor kinetics to redirect ATP to the immunosuppressive adenosine-rich tumor microenvironment (206).

Vascular regeneration of tumor cells enhances the migratory as well as metastatic capacities of tumor cells, in addition to providing them with a favorable nutritional environment (207). Some exosomal proteins promote distant tumor cell metastasis through this process. Gesierich et al. reported that quadruple transmembrane protein-8 (Tspan8)-positive exosomes promoted endothelial cell production and increased the expressions of vascular endothelial growth factors as well as growth factor receptors in fibroblasts, thereby promoting angiogenesis in pancreatic and gastric cancers (208). Chen et al. found that in patients with metastatic colon cancer, high serum Galectin-3 levels were associated with elevated serum G-CSF, IL-6 and sICAM1 levels, which interacts with the vascular endothelium to increase the expressions of vascular cell adhesion protein type I (VCAM-1) on endothelial cell surfaces, leading to increased cancer cell-endothelial adhesion and increased endothelial cell migration and small vessel formation (209). In addition, intramembrane cleavage mediated by γ-secretase, a large protease complex consisting of a catalytic subunit (presenilin-1 or presenilin-2) and auxiliary subunits (Pen-2, Aph1 and nicastrin), is an important link in the Notch signaling pathway. Zeng et al. documented that γ-secretase affects cancer metastasis after Notch activation cascade reactions, probably because γ-secretase promotes angiogenesis in solid tumors through Notch signaling (210).

In conclusion, the establishment of metastatic ecotone, including immunosuppression and angiogenesis suggests that PCaDEPr is involved in mediating the establishment of pre-metastatic ecotone in tumors, thereby inducing cancer metastasis.

### Alterations of Microenvironments at the Target Site

Adaptive regulation of the microenvironment at tumor colonization sites prior to metastasis is important for tumor colonization. Recently, exosomal proteins have been shown to promote tumor cell colonization of tissues and organs by modulating the tumor metastasis target site microenvironments. In addition to alterations of tumor microenvironments at the target site, there are changes in bone colonization processes, such as the number and structures of outcomes and osteoclasts. Prior to the arrival of tumor cells, primary tumors actively regulate the nutritional, extracellular matrix and immune environments of distant organs by secreting regulatory factors, thus producing a permissive and supportive ecological niche for tumor survival at the metastatic site. In tumor bone cell metastasis, malignant communication between PCa cells and bone cells (osteoblasts and osteoclasts) is established. Casimiro et al. found that PCa cells provide osteoblasts with osteogenic cytokines [e.g. bone morphogenetic proteins (BMPs), platelet-derived growth factors (PDGF), endothelin-1 (ET1)] and osteolytic factors [e.g. MMPs and vascular endothelial growth factor (VEGF)], which enables these cells to make bone-derived cell growth factors (211). Itoh et al. identified the ETS Proto-Oncogene 1 (Ets1) protein in PCa-derived exosomes to be an osteoblast differentiation-related transcription factor and found it to be a candidate inducer of osteoblast differentiation (19). A standard exosomal protein study found that exosome-mediated translocation of pyruvate kinase M2 (PKM 2) from PCa cells into BMSCs promotes PCa bone metastasis. Moreover, the PKM2 protein upregulates hypoxia-inducible factor 1α (HIF-1α) in BMSCs to promote CXCL12 expressions in stromal cells. Biologically, exosome-mediated PKM2 transport of prostate tumor origin is a key mediator of PCa bone metastasis (45).

### EMT Transformation and Regulation of Cell Motility

Prior to metastasis, tumor cells are detached from their original sites through loss of attachment and adhesion capacities and metastasize to their target sites with blood or lymphatic chemotaxis, eventually undergoing clonal growth at metastatic sites. Therefore, epithelial-mesenchymal transition processes of EMT formation are important in initiation of cancer metastasis. Various molecules, including exosomal proteins, are involved in EMT transformation in tumor cells. The loss of E-cadherin is associated with the loss of intercellular contacts, disruption of the E-cadherin-catenin complex, abnormal activation of β-catenin signaling as well as cytoskeletal changes. This is critical for cells to lose their epithelial polarity and acquire aggressive phenotypes. In primary PCa, suppressed E-cadherin levels and elevated nucleus β-catenin levels are strongly associated with metastasis and poor prognostic outcomes. Zhang et al. observed elevated E-cadherin levels and suppressed N-cadherin as well as wave protein levels in response to melanopsin depletion. Silencing of melanopsin was associated with suppressed total and activated β-catenin levels. In a subsequent study, it was noted that when melanopsin was downregulated, PCa cells exhibited decreased proliferation, migration and invasion abilities (101). In addition to melanopsin, Rab3D induces epithelial mesenchymal transformation. Tauro et al. found that Rab3D regulates EMT transformation of tumor cells by activating the Akt/GSK-3β/Snail signaling pathways (212). In addition, overexpressing cells with melanopsin-like Rab2A suppresses E-calmodulin while elevating N-calmodulin, wave protein, and fibronectin levels, which affects the EMT phenotype (118).

Cell motility is key in cancer invasion and metastasis. The loss of cell-cell adhesion and enhanced cell-matrix interactions are essential for enhanced tumor cell motility (213). Four-transmembrane proteins are associated with various processes, including signal transduction pathways, cell activation, proliferation, motility, adhesion, tissue differentiation, angiogenesis, tumor progression, and metastasis (214, 215)and are present in urinary exosomes of PCa patients. Even though most tetra-transmembrane proteins are downregulated in metastatic tumors, the CD151 glycoprotein was the first member of the tetra-transmembrane protein to be identified as a metastasis promoter. This shows that the tetra-transmembrane superfamily protein CD151 promotes cancer migration and metastasis (216). Detchokul et al. revealed that CD151 can regulate the redistribution of adhesion components required for cell migration as well as invasion and the process of targeted delivery of matrix degrading enzymes, confirming that CD151 promotes cell motility and tumor invasion (217). Gesierich et al. found that colocalization of integrin β4 with CD151 activates PKC to promote integrin internalization, thereby increasing tumor cell motility (218). Ang et al. found that CD151-transfected LNCaP cells had greater motility, compared to controls and that PC3 cells with CD151 knockdown showed reduced motility. However, the responsible mechanisms have not been elucidated (219). In conclusion, CD151 induces distant cancer cell metastasis by regulating tumor cell motility.

### Activation of Metastatic Signaling Pathways

Tumor-associated exosomal proteins have the ability to mediate the activation of common signaling pathways to induce tumor cell metastasis. Hao et al. found that CD44 or CD147 knockdown downregulated p-Akt and p-Erk levels in PC3 cells and inhibited the activations of PI3K/Akt and MAPK/Erk signaling pathways. The administration of drugs that selectively target CD44/CD147 alone or in combination with docetaxel restricted CaP metastasis (50). Therefore, CD44 and CD147 enhances the metastatic abilities of CaP cells, possibly by activating PI3K and MAPK pathways. He et al. found that DJ-1 knockdown markedly suppressed invasive and migration abilities of pancreatic cancer cells, inhibited the expressions and activities of uPA and induced cytoskeletal disruption. These outcomes may have been because DJ-1 downregulation inhibited SRC and ERK1/2 phosphorylation, which suppressed SRC and ERK signaling pathways-mediated expressions of uPA (220). Yang et al. reported that the exosomal protein (Rab3D) was highly expressed in malignant breast cancer but not in normal tissues and benign breast tumors. The knockdown of Rab3D significantly inhibited the migration abilities of breast cancer cells, which was confirmed to be mediated by Rab3D activations of AKT/GSK-3β/Snail signaling pathways (116). In addition, exosoemal proteins are involved in intermediate pathways of metastatic signaling pathways to induce cancer metastasis. Boscher et al. found that EGF activations of downstream integrin signaling pathways in breast cancer adenocarcinoma epithelial cells induces tumor metastasis dependent on synergistic actions of Galectin 3 and p-Caveolin-1 (221). Thus, PCaDEPr activates multiple tumor metastasis signaling pathways to induce cancer metastasis.

## PCaDEPr in Cancer Drug Resistance

Tumor cell sensitivity to chemotherapeutic agents is essential for cancer drug therapy. Many biological factors modulate the sensitivity as well as resistance of tumors to chemotherapeutic agents (222, 223). In patients with prostate tumors, exosomal proteins have been shown to be essential for the development of drug resistance. With increasing administrations of chemotherapeutic drugs, the rates of tumor drug resistance have been increasing year by year. Therefore, elucidation of the mechanisms involved in chemotherapeutic resistance to identify new therapeutic targets is the direction of today’s oncology research. Previous exosomes studies found that PCaDEPr regulates tumor sensitivity to drugs through various pathways. For example, Survivin is expressed in PCa-derived exosomes and its downregulation sensitizes PCa cells to chemotherapeutic agents (59). Doxorubicin is a chemotherapeutic agent that usually becomes ineffective against tumor cells over time due to chemoresistance. Breast cancer cells lacking LAMP2A exhibit increased sensitivity to this drug (224). In addition, LAMP2-mediated autophagy in PCa-derived exosomes modulates lung cancer cell resistance to temozolomide (225). Pedram et al. found that resistance of DU145 and PC-3 to docetaxel and paclitaxel was partly due to P-gp expressions and confirmed that P-gp protein levels in exosomes reflect P-gp levels in PCa cells (226).

In cisplatin-resistant ovarian cancer cells, claudin-4 was overexpressed 7.2-fold and was one of the most overexpressed proteins, suggesting that it may be associated with cisplatin resistance in ovarian cancer. Expressions of claudin, including claudin-3, -4 and -7, were markedly higher in chemoresistant ovarian cancer cells than in chemo-sensitive ovarian cancer cells. Their high expressions were positively correlated with ovarian cancer resistance to chemotherapy (227). Liu et al. found that elevated levels of synaptic binding protein-like 4 (SYTL4), a Rab effector in vesicular transport, are associated with poor prognostic outcomes in TNBC (triple negative breast cancer, referring to breast cancer lacking estrogen receptor (ESr or Er), progesterone receptor (Pr) expression with lack of epidermal growth factor receptor-2 gene (HER) expression), especially in paclitaxel treated TNBC. It has been postulated that SYTL4 confers resistance to paclitaxel in triple-negative breast cancer (110).

These findings demonstrate that PCaDEPr plays an important role in promoting drug resistance in tumor cells.

## Summary and Outlook

With further research on PCaDE, tumor-derived exosomal proteins have attracted special attention. In this review, we discuss recent advances in research related to PCaDEPrs from the perspective of promoting tumorigenesis and progression. The role of these exosomal proteins present in cells or other tumors is also highlighted, although this does not mean that they remain such in specific tumor exosomes. However, because of this, this may provide researchers who identify differential proteins by routine protein analysis for subsequent functional validation with new directions for these exosomal proteins in prostate cancer research.

Although PSA is of great value as a commonly used tumor marker in the diagnosis and prognosis of prostate cancer, it has undeniable limitations, especially for the early diagnosis of bone metastatic prostate cancer. Exosomes may have more potential than PSA for therapeutic purposes, with a number of publications reporting that interference with exosome production and expression of exosome-containing substances will significantly reduce tumor metastasis and aggressiveness. In addition, important progress has been made in the study of drug-loaded exosomes, modified exosomes, and MSC exosomes in disease therapy. However, several questions remain to be addressed in future studies:1. With the study of exosome proteomics, more and more different kinds of proteins have been discovered one after another. However, it is not possible to conclude that the extracted proteins are necessarily present in exosomes according to the current database, so a more rigorous and extensive study is still needed to clarify the types of substances contained in tumor-derived exosomes in order to exclude heterogeneous proteins. 2. Due to the limitations of current extraction techniques, it is difficult to extract exosomes with 100% purity, and exosomes themselves contain a variety of secretory proteins, so it is difficult to determine the exact source of secretory proteins in exosomes of somatic fluid origin: exosomal origin? Body fluids themselves contain? 3. We found that tumors can release some exosomes rich in protective proteins that can inhibit cancer progression, so extracting these exosomes for interfering with tumor progression may be a new avenue for tumor therapy. 4. A large number of studies have found that some proteins present in exosomes and with protective effects significantly decrease with cancer progression. it remains unclear whether the effect of exosomes derived from primary and bone metastatic PCa on the establishment of the target microenvironment is persistent or transient, and further studies of these exosomes are therefore still necessary.

Bone metastatic prostate cancer and the emergence of CRPC types pose great difficulties in the treatment of PCa. Recent literature has demonstrated that tumor-derived exosomal proteins can be transported to distant metastatic targets, creating “fertile ground” to promote cancer metastasis. This may offer hope for finding ways to diagnose and treat bone metastases from prostate cancer. Furthermore, exploring the role of tumor-derived exosomes in cancer development may be a way to address these challenges. The successful treatment of these complex cancers depends on our full understanding of the single actions or interactions and mechanisms of action of the various components of exocytosis. We elucidated on the various functions and possible mechanisms of exosomeal proteins in PCa body fluids or tissues during tumor development. The exosomeal proteins can influence tumor initiation, progression, and drug resistance processes through various complex mechanisms. Elucidation of the mechanisms through which biomolecules, such as proteins, act on these processes will make it possible for us to target these proteins for cancer treatment. However, the most suitable exosomes molecular target for the diagnosis and treatment of PCa has yet to be identified, and the clinical applications of exosomes are associated with some challenges. For instance, exosomes isolation and extraction methods are still limited to the laboratory, relatively harsh storage conditions for exosomes, and medical costs. With rapid advances in exosome-related technologies and in-depth research on PCaDEPr, applications of exosomal proteins in the diagnosis and treatment of PCa will soon be realized.

## Author Contributions

SF and KL searched for literature and wrote the first draft of this article. SF edited tables and figures. JZ and GZ reviewed the manuscript and polished the grammar. All authors approved the final version submitted and agree on its submission to this journal.

## Funding

This work was supported by the National Natural Science Foundation of China (grant nos. 81760462 and 81860456).

## Conflict of Interest

The authors declare that the research was conducted in the absence of any commercial or financial relationships that could be construed as a potential conflict of interest.

## Publisher’s Note

All claims expressed in this article are solely those of the authors and do not necessarily represent those of their affiliated organizations, or those of the publisher, the editors and the reviewers. Any product that may be evaluated in this article, or claim that may be made by its manufacturer, is not guaranteed or endorsed by the publisher.

## References

[1] Lorenc T Klimczyk K Michalczewska I Słomka M Kubiak-Tomaszewska G Olejarz W. Exosomes in Prostate Cancer Diagnosis, Prognosis and Therapy. Int J Mol Sci (2020) 21(6):2118. doi: 10.3390/ijms21062118 PMC7139716 32204455

[2] Liu CM Hsieh CL Shen CN Lin CC Shigemura K Sung SY. Exosomes From the Tumor Microenvironment as Reciprocal Regulators That Enhance Prostate Cancer Progression. Int J Urol (2016) 23(9):734–44. doi: 10.1111/iju.13145 27397852

[3] Guo T Wang Y Jia J Mao X Stankiewicz E Scandura G. The Identification of Plasma Exosomal miR-423-3p as a Potential Predictive Biomarker for Prostate Cancer Castration-Resistance Development by Plasma Exosomal miRNA Sequencing. Front Cell Dev Biol (2020) 8:602493. doi: 10.3389/fcell.2020.602493 33490068 PMC7817948

[4] Dragomir M Chen B Calin GA. Exosomal lncRNAs as New Players in Cell-to-Cell Communication. Transl Cancer Res (2018) 7(Suppl 2):S243–s252. doi: 10.21037/tcr.2017.10.46 30148073 PMC6107076

[5] Stade K Ford CS Guthrie C Weis K. Exportin 1 (Crm1p) is an Essential Nuclear Export Factor. Cell (1997) 90(6):1041–50. doi: 10.1016/S0092-8674(00)80370-0 9323132

[6] Duijvesz D Burnum-Johnson KE Gritsenko MA Hoogland AM Vredenbregt-van den Berg MS Willemsen R. Proteomic Profiling of Exosomes Leads to the Identification of Novel Biomarkers for Prostate Cancer. PloS One (2013) 8(12):e82589. doi: 10.1371/journal.pone.0082589 24391718 PMC3876995

[7] Kowal J Tkach M Théry C. Biogenesis and Secretion of Exosomes. Curr Opin Cell Biol (2014) 29:116–25. doi: 10.1016/j.ceb.2014.05.004 24959705

[8] Kowal J Arras G Colombo M Jouve M Morath JP Primdal-Bengtson B. Proteomic Comparison Defines Novel Markers to Characterize Heterogeneous Populations of Extracellular Vesicle Subtypes. Proc Natl Acad Sci USA (2016) 113(8):E968–77. doi: 10.1073/pnas.1521230113 PMC4776515 26858453

[9] Andreu Z Yáñez-Mó M. Tetraspanins in Extracellular Vesicle Formation and Function. Front Immunol (2014) 5:442. doi: 10.3389/fimmu.2014.00442 25278937 PMC4165315

[10] Shahir M Mahmoud Hashemi S Asadirad A Varahram M Kazempour-Dizaji M Folkerts G. Effect of Mesenchymal Stem Cell-Derived Exosomes on the Induction of Mouse Tolerogenic Dendritic Cells. J Cell Physiol (2020) 235(10):7043–55. doi: 10.1002/jcp.29601 PMC7496360 32043593

[11] Huang D Chen J Hu D Xie F Yang T Li Z. Advances in Biological Function and Clinical Application of Small Extracellular Vesicle Membrane Proteins. Front Oncol (2021) 11:675940. doi: 10.3389/fonc.2021.675940 34094979 PMC8172959

[12] Fhu CW Ali A. Fatty Acid Synthase: An Emerging Target in Cancer. Mol (Basel Switzerland) (2020) 25(17):3935. doi: 10.3390/molecules25173935 PMC7504791 32872164

[13] Azizian NG Li Y. XPO1-Dependent Nuclear Export as a Target for Cancer Therapy. J Hematol Oncol (2020) 13(1):61. doi: 10.1186/s13045-020-00903-4 32487143 PMC7268335

[14] Li HJ Ke FY Lin CC Lu MY Kuo YH Wang YP. ENO1 Promotes Lung Cancer Metastasis *via* HGFR and WNT Signaling-Driven Epithelial-To-Mesenchymal Transition. Cancer Res (2021) 81(15):4094–109. doi: 10.1158/0008-5472.CAN-20-3543 34145039

[15] Husi H Skipworth RJ Cronshaw A Stephens NA Wackerhage H Greig C. Programmed Cell Death 6 Interacting Protein (PDCD6IP) and Rabenosyn-5 (ZFYVE20) are Potential Urinary Biomarkers for Upper Gastrointestinal Cancer. Proteomics Clin Appl (2015) 9(5-6):586–96. doi: 10.1002/prca.201400111 25644331

[16] Kurozumi A Goto Y Matsushita R Fukumoto I Kato M Nishikawa R. Tumor-Suppressive microRNA-223 Inhibits Cancer Cell Migration and Invasion by Targeting ITGA3/ITGB1 Signaling in Prostate Cancer. Cancer Sci (2016) 107(1):84–94. doi: 10.1111/cas.12842 26509963 PMC4724812

[17] Pellinen T Blom S Sánchez S Välimäki K Mpindi JP Azegrouz H. ITGB1-Dependent Upregulation of Caveolin-1 Switches Tgfβ Signalling From Tumour-Suppressive to Oncogenic in Prostate Cancer. Sci Rep (2018) 8(1):2338. doi: 10.1038/s41598-018-20161-2 29402961 PMC5799174

[18] Kato T Mizutani K Kameyama K Kawakami K Fujita Y Nakane K. Serum Exosomal P-Glycoprotein is a Potential Marker to Diagnose Docetaxel Resistance and Select a Taxoid for Patients With Prostate Cancer. Urol Oncol (2015) 33(9):385.e15–20. doi: 10.1016/j.urolonc.2015.04.019 26027763

[19] Itoh T Ito Y Ohtsuki Y Ando M Tsukamasa Y Yamada N. Microvesicles Released From Hormone-Refractory Prostate Cancer Cells Facilitate Mouse Pre-Osteoblast Differentiation. J Mol Histol (2012) 43(5):509–15. doi: 10.1007/s10735-012-9415-1 PMC3460166 22526510

[20] Wan X Kim SY Guenther LM Mendoza A Briggs J Yeung C. Beta4 Integrin Promotes Osteosarcoma Metastasis and Interacts With Ezrin. Oncogene (2009) 28(38):3401–11. doi: 10.1038/onc.2009.206 PMC2753583 19597468

[21] Kawakami K Fujita Y Kato T Mizutani K Kameyama K Tsumoto H. Integrin β4 and Vinculin Contained in Exosomes are Potential Markers for Progression of Prostate Cancer Associated With Taxane-Resistance. Int J Oncol (2015) 47(1):384–90. doi: 10.3892/ijo.2015.3011 25997717

[22] Hosseini-Beheshti E Pham S Adomat H Li N Tomlinson Guns ES. Exosomes as Biomarker Enriched Microvesicles: Characterization of Exosomal Proteins Derived From a Panel of Prostate Cell Lines With Distinct AR Phenotypes. Mol Cell Proteomics (2012) 11(10):863–85. doi: 10.1074/mcp.M111.014845 PMC3494141 22723089

[23] Chen CY Lin YS Chen CH Chen YJ. Annexin A2-Mediated Cancer Progression and Therapeutic Resistance in Nasopharyngeal Carcinoma. J Biomed Sci (2018) 25(1):30. doi: 10.1186/s12929-018-0430-8 29598816 PMC5877395

[24] Chu Y Lai YH Lee MC Yeh YJ Wu YK Tsao W. Calsyntenin-1, Clusterin and Neutrophil Gelatinase-Associated Lipocalin are Candidate Serological Biomarkers for Lung Adenocarcinoma. Oncotarget (2017) 8(64):107964–76. doi: 10.18632/oncotarget.22438 PMC5746118 29296216

[25] Watt F Martorana A Brookes DE Ho T Kingsley E O'Keefe DS. A Tissue-Specific Enhancer of the Prostate-Specific Membrane Antigen Gene, FOLH1. Genomics (2001) 73(3):243–54. doi: 10.1006/geno.2000.6446 11350116

[26] Li S Ma YM Zheng PS Zhang P. GDF15 Promotes the Proliferation of Cervical Cancer Cells by Phosphorylating AKT1 and Erk1/2 Through the Receptor Erbb2. J Exp Clin Cancer Res CR (2018) 37(1):80. doi: 10.1186/s13046-018-0744-0 29636108 PMC5894198

[27] Kwon HJ Min SY Park MJ Lee C Park JH Chae JY. Expression of CD9 and CD82 in Clear Cell Renal Cell Carcinoma and its Clinical Significance. Pathol Res Pract (2014) 210(5):285–90. doi: 10.1016/j.prp.2014.01.004 24553302

[28] Mizutani K Terazawa R Kameyama K Kato T Horie K Tsuchiya T. Isolation of Prostate Cancer-Related Exosomes. Anticancer Res (2014) 34(7):3419–23. 24982349

[29] Yang XF Wu CJ Chen L Alyea EP Canning C Kantoff P. CML28 is a Broadly Immunogenic Antigen, Which is Overexpressed In Tumor Cells. Cancer Res (2002) 62(19):5517–22. PMC4762257 12359762

[30] Fedele C Singh A Zerlanko BJ Iozzo RV Languino LR. The αvβ6 Integrin is Transferred Intercellularly *via* Exosomes. J Biol Chem (2015) 290(8):4545–51. doi: 10.1074/jbc.C114.617662 PMC4335196 25568317

[31] Trerotola M Ganguly KK Fazli L Fedele C Lu H Dutta A. Trop-2 is Up-Regulated in Invasive Prostate Cancer and Displaces FAK From Focal Contacts. Oncotarget (2015) 6(16):14318–28. doi: 10.18632/oncotarget.3960 PMC4546469 26015409

[32] Kumar D Gupta D Shankar S Srivastava RK. Biomolecular Characterization of Exosomes Released From Cancer Stem Cells: Possible Implications for Biomarker and Treatment of Cancer. Oncotarget (2015) 6(5):3280–91. doi: 10.18632/oncotarget.2462 PMC4413653 25682864

[33] Zhu C Kong Z Wang B Cheng W Wu A Meng X. ITGB3/CD61: A Hub Modulator and Target in the Tumor Microenvironment. Am J Trans Res (2019) 11(12):7195–208. PMC6943458 31934272

[34] Vences-Catalán F Duault C Kuo CC Rajapaksa R Levy R Levy S. CD81 as a Tumor Target. Biochem Soc Trans (2017) 45(2):531–5. doi: 10.1042/BST20160478 28408492

[35] Miyata Y Nakamoto H Neckers L. The Therapeutic Target Hsp90 and Cancer Hallmarks. Curr Pharm Design (2013) 19(3):347–65. doi: 10.2174/138161213804143725 PMC7553218 22920906

[36] Albakova Z Armeev GA Kanevskiy LM Kovalenko EI Sapozhnikov AM. HSP70 Multi-Functionality in Cancer. Cells (2020) 9(3):587. doi: 10.3390/cells9030587 PMC7140411 32121660

[37] Wang T Wang Z Niu R Wang L. Crucial Role of Anxa2 in Cancer Progression: Highlights on its Novel Regulatory Mechanism. Cancer Biol Med (2019) 16(4):671–87. doi: 10.20892/j.issn.2095-3941.2019.0228 PMC6936236 31908887

[38] Ramteke A Ting H Agarwal C Mateen S Somasagara R Hussain A. Exosomes Secreted Under Hypoxia Enhance Invasiveness and Stemness of Prostate Cancer Cells by Targeting Adherens Junction Molecules. Mol Carcinog (2015) 54(7):554–65. doi: 10.1002/mc.22124 PMC4706761 24347249

[39] Colak S Ten Dijke P. Targeting TGF-β Signaling in Cancer. Trends Cancer (2017) 3(1):56–71. doi: 10.1016/j.trecan.2016.11.008 28718426

[40] Abd Elmageed ZY Yang Y Thomas R Ranjan M Mondal D Moroz K. Neoplastic Reprogramming of Patient-Derived Adipose Stem Cells by Prostate Cancer Cell-Associated Exosomes. Stem Cells (2014) 32(4):983–97. doi: 10.1002/stem.1619 PMC4184251 24715691

[41] Liu S Liang J Liu Z Zhang C Wang Y Watson AH. The Role of CD276 in Cancers. Front Oncol (2021) 11:654684. doi: 10.3389/fonc.2021.654684 33842369 PMC8032984

[42] Lu Q. δ-Catenin Dysregulation in Cancer: Interactions With E-Cadherin and Beyond. J Pathol (2010) 222(2):119–23. doi: 10.1002/path.2755 PMC2935513 20715154

[43] Lu Q Zhang J Allison R Gay H Yang WX Bhowmick NA. Identification of Extracellular Delta-Catenin Accumulation for Prostate Cancer Detection. Prostate (2009) 69(4):411–8. doi: 10.1002/pros.20902 PMC2633034 19116988

[44] Feng Y Xiong Y Qiao T Li X Jia L Han Y. A Key Player in Carcinogenesis and Potential Target in Cancer Therapy. Cancer Med (2018) 7(12):6124–36. doi: 10.1002/cam4.1820 PMC6308051 30403008

[45] Dai J Escara-Wilke J Keller JM Jung Y Taichman RS Pienta KJ. Primary Prostate Cancer Educates Bone Stroma Through Exosomal Pyruvate Kinase M2 to Promote Bone Metastasis. J Exp Med (2019) 216(12):2883–99. doi: 10.1084/jem.20190158 PMC6888980 31548301

[46] Agarwal R D'Souza T Morin PJ. Claudin-3 and Claudin-4 Expression in Ovarian Epithelial Cells Enhances Invasion and is Associated With Increased Matrix Metalloproteinase-2 Activity. Cancer Res (2005) 65(16):7378–85. doi: 10.1158/0008-5472.CAN-05-1036 16103090

[47] Lin X Shang X Manorek G Howell SB. Regulation of the Epithelial-Mesenchymal Transition by Claudin-3 and Claudin-4. PLoS One (2013) 8(6):e67496. doi: 10.1371/journal.pone.0067496 23805314 PMC3689737

[48] Kharaziha P Chioureas D Rutishauser D Baltatzis G Lennartsson L Fonseca P. Molecular Profiling of Prostate Cancer Derived Exosomes may Reveal a Predictive Signature for Response to Docetaxel. Oncotarget (2015) 6(25):21740–54. doi: 10.18632/oncotarget.3226 PMC4673300 25844599

[49] Díaz MI Díaz P Bennett JC Urra H Ortiz R Orellana PC. Caveolin-1 Suppresses Tumor Formation Through the Inhibition of the Unfolded Protein Response. Cell Death Dis (2020) 11(8):648. doi: 10.1038/s41419-020-02792-4 32811828 PMC7434918

[50] Hao J Madigan MC Khatri A Power CA Hung TT Beretov J. *In Vitro* and *In Vivo* Prostate Cancer Metastasis and Chemoresistance can be Modulated by Expression of Either CD44 or CD147. PLoS One (2012) 7(8):e40716. doi: 10.1371/journal.pone.0040716 22870202 PMC3411712

[51] Xiao Y Zhong J Zhong B Huang J Jiang L Jiang Y. Exosomes as Potential Sources of Biomarkers in Colorectal Cancer. Cancer Lett (2020) 476:13–22. doi: 10.1016/j.canlet.2020.01.033 32044357

[52] Ishizuya Y Uemura M Narumi R Tomiyama E Koh Y Matsushita M. The Role of Actinin-4 (ACTN4) in Exosomes as a Potential Novel Therapeutic Target in Castration-Resistant Prostate Cancer. Biochem Biophys Res Commun (2020) 523(3):588–94. doi: 10.1016/j.bbrc.2019.12.084 31941606

[53] Khurana S Chakraborty S Lam M Liu Y Su YT Zhao X. Familial Focal Segmental Glomerulosclerosis (FSGS)-Linked α-Actinin 4 (ACTN4) Protein Mutants Lose Ability to Activate Transcription by Nuclear Hormone Receptors. J Biol Chem (2012) 287(15):12027–35. doi: 10.1074/jbc.M112.345421 PMC3320949 22351778

[54] Bergelson LD. Serum Gangliosides as Endogenous Immunomodulators. Immunol Today (1995) 16(10):483–6. doi: 10.1016/0167-5699(95)80032-8 7576052

[55] Hata K Tochigi T Sato I Kawamura S Shiozaki K Wada T. Increased Sialidase Activity in Serum of Cancer Patients: Identification of Sialidase and Inhibitor Activities in Human Serum. Cancer Sci (2015) 106(4):383–9. doi: 10.1111/cas.12627 PMC4409881 25652216

[56] Locke JA Fazli L Adomat H Smyl J Weins K Lubik AA. A Novel Communication Role for CYP17A1 in the Progression of Castration-Resistant Prostate Cancer. Prostate (2009) 69(9):928–37. doi: 10.1002/pros.20940 19267349

[57] Hurwitz MD Kaur P Nagaraja GM Bausero MA Manola J Asea A. Radiation Therapy Induces Circulating Serum Hsp72 in Patients With Prostate Cancer. Radiother Oncol (2010) 95(3):350–8. doi: 10.1016/j.radonc.2010.03.024 PMC2883632 20430459

[58] Kishi H Igawa M Kikuno N Yoshino T Urakami S Shiina H. Expression of the Survivin Gene in Prostate Cancer: Correlation With Clinicopathological Characteristics, Proliferative Activity and Apoptosis. J Urol (2004) 171(5):1855–60. doi: 10.1097/01.ju.0000120317.88372.03 15076293

[59] Khan S Jutzy JM Valenzuela MM Turay D Aspe JR Ashok A. Plasma-Derived Exosomal Survivin, A Plausible Biomarker for Early Detection of Prostate Cancer. PLoS One (2012) 7(10):e46737. doi: 10.1371/journal.pone.0046737 23091600 PMC3473028

[60] Krishn SR Singh A Bowler N Duffy AN Friedman A Fedele C. Prostate Cancer Sheds the αvβ3 Integrin *In Vivo* Through Exosomes. Matrix Biol (2019) 77:41–57. doi: 10.1016/j.matbio.2018.08.004 30098419 PMC6541230

[61] Kwon MJ. Emerging Roles of Claudins in Human Cancer. Int J Mol Sci (2013) 14(9):18148–80. doi: 10.3390/ijms140918148 PMC3794774 24009024

[62] Gagou ME Ganesh A Thompson R Phear G Sanders C Meuth M. Suppression of Apoptosis by PIF1 Helicase in Human Tumor Cells. Cancer Res (2011) 71(14):4998–5008. doi: 10.1158/0008-5472.CAN-10-4404 21616935

[63] Ding L Wang Z Yan J Yang X Liu A Qiu W. Human Four-and-a-Half LIM Family Members Suppress Tumor Cell Growth Through a TGF-Beta-Like Signaling Pathway. J Clin Invest (2009) 119(2):349–61. doi: 10.1172/JCI35930 PMC2631293 19139564

[64] Terayama M Yamada K Hagiwara T Inazuka F Sezaki T Igari T. Glutathione S-Transferase Omega 2 Regulates Cell Growth and the Expression of E-Cadherin *via* Post-Transcriptional Down-Regulation of β-Catenin in Human Esophageal Squamous Cells. Carcinogenesis (2020) 41(7):875–86. doi: 10.1093/carcin/bgz189 31738399

[65] Bolomsky A Heusschen R Schlangen K Stangelberger K Muller J Schreiner W. Maternal Embryonic Leucine Zipper Kinase is a Novel Target for Proliferation-Associated High-Risk Myeloma. Haematologica (2018) 103(2):325–35. doi: 10.3324/haematol.2017.172973 PMC5792277 29122991

[66] Barrios N González-Pérez E Hernández R Campuzano S. The Homeodomain Iroquois Proteins Control Cell Cycle Progression and Regulate the Size of Developmental Fields. PLoS Genet (2015) 11(8):e1005463. doi: 10.1371/journal.pgen.1005463 26305360 PMC4549242

[67] Berezovsky AD Poisson LM Cherba D Webb CP Transou AD Lemke NW. Sox2 Promotes Malignancy in Glioblastoma by Regulating Plasticity and Astrocytic Differentiation. Neoplasia (New York N.Y.) (2014) 16(3):193–206, 206.e19-25. doi: 10.1016/j.neo.2014.03.006 PMC4094829 24726753

[68] Lei M. The MCM Complex: Its Role in DNA Replication and Implications for Cancer Therapy. Curr Cancer Drug Targets (2005) 5(5):365–80. doi: 10.2174/1568009054629654 16101384

[69] Sim EU Lee CW Narayanan K. The Roles of Ribosomal Proteins in Nasopharyngeal Cancer: Culprits, Sentinels or Both. biomark Res (2021) 9(1):51. doi: 10.1186/s40364-021-00311-x 34193301 PMC8247250

[70] Patnaik D Estève PO Pradhan S. Targeting the SET and RING-Associated (SRA) Domain of Ubiquitin-Like, PHD and Ring Finger-Containing 1 (UHRF1) for Anti-Cancer Drug Development. Oncotarget (2018) 9(40):26243–58. doi: 10.18632/oncotarget.25425 PMC5995235 29899856

[71] Walsh PC. Heterogeneity of Genetic Alterations in Prostate Cancer: Evidence of the Complex Nature of the Disease. J Urol (2002) 168(4 Pt 1):1635–6. 12356045

[72] Liu Z Johnson ST Zhang Z Corey DR. Expression of TNRC6 (GW182) Proteins Is Not Necessary for Gene Silencing by Fully Complementary RNA Duplexes. Nucleic Acid Ther (2019) 29(6):323–34. doi: 10.1089/nat.2019.0815 PMC6885777 31670606

[73] Anand R Prakash SS Veeramanikandan R Kirubakaran R. Association Between Apolipoprotein E Genotype and Cancer Susceptibility: A Meta-Analysis. J Cancer Res Clin Oncol (2014) 140(7):1075–85. doi: 10.1007/s00432-014-1634-2 PMC11823783 24706182

[74] Welton JL Brennan P Gurney M Webber JP Spary LK Carton DG. Proteomics Analysis of Vesicles Isolated From Plasma and Urine of Prostate Cancer Patients Using a Multiplex, Aptamer-Based Protein Array. J Extracell Vesicles (2016) 5:31209. doi: 10.3402/jev.v5.31209 27363484 PMC4929354

[75] Afshar-Kharghan V. The Role of the Complement System in Cancer. J Clin Invest (2017) 127(3):780–9. doi: 10.1172/JCI90962 PMC5330758 28248200

[76] Mangogna A Belmonte B Agostinis C Zacchi P Iacopino DG Martorana A. Prognostic Implications of the Complement Protein C1q in Gliomas. Front Immunol (2019) 10:2366. doi: 10.3389/fimmu.2019.02366 31649675 PMC6795702

[77] Riihilä P Viiklepp K Nissinen L Farshchian M Kallajoki M Kivisaari A. Tumour-Cell-Derived Complement Components C1r and C1s Promote Growth of Cutaneous Squamous Cell Carcinoma. Br J Dermatol (2020) 182(3):658–70. doi: 10.1111/bjd.18095 PMC7065064 31049937

[78] Dirix LY Salgado R Weytjens R Colpaert C Benoy I Huget P. Plasma Fibrin D-Dimer Levels Correlate With Tumour Volume, Progression Rate and Survival in Patients With Metastatic Breast Cancer. Br J Cancer (2002) 86(3):389–95. doi: 10.1038/sj.bjc.6600069 PMC2375200 11875705

[79] Simpson-Haidaris PJ Rybarczyk B. Tumors and Fibrinogen. The Role of Fibrinogen as an Extracellular Matrix Protein. Ann New York Acad Sci (2001) 936:406–25. doi: 10.1111/j.1749-6632.2001.tb03525.x 11460495

[80] Lin TC Yang CH Cheng LH Chang WT Lin YR Cheng HC. Fibronectin in Cancer: Friend or Foe. Cells (2019) 9(1):27. doi: 10.3390/cells9010027 PMC7016990 31861892

[81] Mangogna A Varghese PM Agostinis C Alrokayan SH Khan HA Stover CM. Prognostic Value of Complement Properdin in Cancer. Front Immunol (2020) 11:614980. doi: 10.3389/fimmu.2020.614980 33542722 PMC7851055

[82] Yang AJ Wang M Wang Y Cai W Li Q Zhao TT. Cancer Cell-Derived Von Willebrand Factor Enhanced Metastasis of Gastric Adenocarcinoma. Oncogenesis (2018) 7(1):12. doi: 10.1038/s41389-017-0023-5 29362409 PMC5833464

[83] Gabriel K Ingram A Austin R Kapoor A Tang D Majeed F. Regulation of the Tumor Suppressor PTEN Through Exosomes: A Diagnostic Potential for Prostate Cancer. PloS One (2013) 8(7):e70047. doi: 10.1371/journal.pone.0070047 23936141 PMC3723640

[84] Frank MH Denton MD Alexander SI Khoury SJ Sayegh MH Briscoe DM. Specific MDR1 P-Glycoprotein Blockade Inhibits Human Alloimmune T Cell Activation *In Vitro* . J Immunol (Baltimore Md. 1950) (2001) 166(4):2451–9. doi: 10.4049/jimmunol.166.4.2451 11160305

[85] Cory TJ He H Winchester LC Kumar S Fletcher CV. Alterations in P-Glycoprotein Expression and Function Between Macrophage Subsets. Pharm Res (2016) 33(11):2713–21. doi: 10.1007/s11095-016-1998-x PMC5042840 27431863

[86] Øverbye A Skotland T Koehler CJ Thiede B Seierstad T Berge V. Identification of Prostate Cancer Biomarkers in Urinary Exosomes. Oncotarget (2015) 6(30):30357–76. doi: 10.18632/oncotarget.4851 PMC4745805 26196085

[87] Bhardwaj A Singh S Srivastava SK Arora S Hyde SJ Andrews J. Restoration of PPP2CA Expression Reverses Epithelial-to-Mesenchymal Transition and Suppresses Prostate Tumour Growth and Metastasis in an Orthotopic Mouse Model. Br J Cancer (2014) 110(8):2000–10. doi: 10.1038/bjc.2014.141 PMC3992501 24642616

[88] Shaughnessy R Echard A. Rab35 GTPase and Cancer: Linking Membrane Trafficking to Tumorigenesis. Traffic (Copenhagen Denmark) (2018) 19(4):247–52. doi: 10.1111/tra.12546 29314576

[89] Nedjadi T Kitteringham N Campbell F Jenkins RE Park BK Navarro P. S100A6 Binds to Annexin 2 in Pancreatic Cancer Cells and Promotes Pancreatic Cancer Cell Motility. Br J Cancer (2009) 101(7):1145–54. doi: 10.1038/sj.bjc.6605289 PMC2768105 19724273

[90] Di Virgilio F. Purines, Purinergic Receptors, and Cancer. Cancer Res (2012) 72(21):5441–7. doi: 10.1158/0008-5472.CAN-12-1600 23090120

[91] Hsu DK Chen HY Liu FT. Galectin-3 Regulates T-Cell Functions. Immunol Rev (2009) 230(1):114–27. doi: 10.1111/j.1600-065X.2009.00798.x 19594632

[92] Nakahara S Oka N Raz A. On the Role of Galectin-3 in Cancer Apoptosis. Apoptosis an Int J Programmed Cell Death (2005) 10(2):267–75. doi: 10.1007/s10495-005-0801-y 15843888

[93] Funasaka T Raz A Nangia-Makker P. Galectin-3 in Angiogenesis and Metastasis. Glycobiology (2014) 24(10):886–91. doi: 10.1093/glycob/cwu086 PMC4153760 25138305

[94] Merseburger AS Kramer MW Hennenlotter J Simon P Knapp J Hartmann JT. Involvement of Decreased Galectin-3 Expression in the Pathogenesis and Progression of Prostate Cancer. Prostate (2008) 68(1):72–7. doi: 10.1002/pros.20688 18008332

[95] Gauthier-Rouvière C Bodin S Comunale F Planchon D. Flotillin Membrane Domains in Cancer. Cancer Metastasis Rev (2020) 39(2):361–74. doi: 10.1007/s10555-020-09873-y PMC7311376 32297092

[96] Cifuentes E Mataraza JM Yoshida BA Menon M Sacks DB Barrack ER. Physical and Functional Interaction of Androgen Receptor With Calmodulin in Prostate Cancer Cells. Proc Natl Acad Sci USA (2004) 101(2):464–9. doi: 10.1073/pnas.0307161101 PMC327170 14695896

[97] Liang H Xiong Z Li R Hu K Cao M Yang J. BDH2 is Downregulated in Hepatocellular Carcinoma and Acts as a Tumor Suppressor Regulating Cell Apoptosis and Autophagy. J Cancer (2019) 10(16):3735–45. doi: 10.7150/jca.32022 PMC6636298 31333791

[98] Zhang SF Wang XY Fu ZQ Peng QH Zhang JY Ye F. TXNDC17 Promotes Paclitaxel Resistance *via* Inducing Autophagy in Ovarian Cancer. Autophagy (2015) 11(2):225–38. doi: 10.1080/15548627.2014.998931 PMC4502659 25607466

[99] Wu Y Du H Zhan M Wang H Chen P Du D. Sepiapterin Reductase Promotes Hepatocellular Carcinoma Progression *via* FoxO3a/Bim Signaling in a Nonenzymatic Manner. Cell Death Dis (2020) 11(4):248. doi: 10.1038/s41419-020-2471-7 32312975 PMC7170898

[100] Zhang X Chen Y Wang K Tang J Chen Y Jin G. The Knockdown of the Sepiapterin Reductase Gene Suppresses the Proliferation of Breast Cancer by Inducing ROS-Mediated Apoptosis. Int J Clin Exp Pathol (2020) 13(9):2228–39. PMC7539867 33042327

[101] Zhang T Sun Y Zheng T Wang R Jia D Zhang W. MLPH Accelerates the Epithelial-Mesenchymal Transition in Prostate Cancer. Onco Targets Ther (2020) 13:701–8. doi: 10.2147/OTT.S225023 PMC6986253 32158222

[102] Wei CY Zhu MX Lu NH Peng R Yang X Zhang PF. Bioinformatics-Based Analysis Reveals Elevated MFSD12 as a Key Promoter of Cell Proliferation and a Potential Therapeutic Target in Melanoma. Oncogene (2019) 38(11):1876–91. doi: 10.1038/s41388-018-0531-6 PMC6462865 30385854

[103] Gonzalez A Valeiras M Sidransky E Tayebi N. Lysosomal Integral Membrane Protein-2: A New Player in Lysosome-Related Pathology. Mol Genet Metab (2014) 111(2):84–91. doi: 10.1016/j.ymgme.2013.12.005 24389070 PMC3924958

[104] Li D Cheng X Zheng W Chen J. Glucosamine-6-Phosphate Isomerase 1 Promotes Tumor Progression and Indicates Poor Prognosis in Hepatocellular Carcinoma. Cancer Manag Res (2020) 12:4923–35. doi: 10.2147/CMAR.S250094 PMC7321694 32606980

[105] Moriwaki K Shinzaki S Miyoshi E. GDP-Mannose-4,6-Dehydratase (GMDS) Deficiency Renders Colon Cancer Cells Resistant to Tumor Necrosis Factor-Related Apoptosis-Inducing Ligand (TRAIL) Receptor- and CD95-Mediated Apoptosis by Inhibiting Complex II Formation. J Biol Chem (2011) 286(50):43123–33. doi: 10.1074/jbc.M111.262741 PMC3234837 22027835

[106] Li Z Xuan W Huang L Chen N Hou Z Lu B. Claudin 10 Acts as a Novel Biomarker for the Prognosis of Patients With Ovarian Cancer. Oncol Lett (2020) 20(1):373–81. doi: 10.3892/ol.2020.11557 PMC7285858 32565963

[107] Andrijes R Hejmadi RK Pugh M Rajesh S Novitskaya V Ibrahim M. Tetraspanin 6 is a Regulator of Carcinogenesis in Colorectal Cancer. Proc Natl Acad Sci USA (2021) 118(39):e2011411118. doi: 10.1073/pnas.2011411118 34521767 PMC8488650

[108] Shih Ie M Wang TL. Notch Signaling, Gamma-Secretase Inhibitors, and Cancer Therapy. Cancer Res (2007) 67(5):1879–82. doi: 10.1158/0008-5472.CAN-06-3958 17332312

[109] Wu PH Onodera Y Giaccia AJ Le QT Shimizu S Shirato H. Lysosomal Trafficking Mediated by Arl8b and BORC Promotes Invasion of Cancer Cells That Survive Radiation. Commun Biol (2020) 3(1):620. doi: 10.1038/s42003-020-01339-9 33110168 PMC7591908

[110] Liu XY Jiang W Ma D Ge LP Yang YS Gou ZC. SYTL4 Downregulates Microtubule Stability and Confers Paclitaxel Resistance in Triple-Negative Breast Cancer. Theranostics (2020) 10(24):10940–56. doi: 10.7150/thno.45207 PMC7532662 33042263

[111] Arumugam T Logsdon CD. S100P: A Novel Therapeutic Target for Cancer. Amino Acids (2011) 41(4):893–9. doi: 10.1007/s00726-010-0496-4 PMC4041611 20509035

[112] Kim RH Peters M Jang Y Shi W Pintilie M Fletcher GC. DJ-1, a Novel Regulator of the Tumor Suppressor PTEN. Cancer Cell (2005) 7(3):263–73. doi: 10.1016/j.ccr.2005.02.010 15766664

[113] Scarl RT Lawrence CM Gordon HM Nunemaker CS. STEAP4: Its Emerging Role in Metabolism and Homeostasis of Cellular Iron and Copper. J Endocrinol (2017) 234(3):R123–r134. doi: 10.1530/JOE-16-0594 28576871 PMC6166870

[114] Zou P Yang Y Xu X Liu B Mei F You J. Silencing of Vacuolar ATPase C Subunit ATP6V0C Inhibits the Invasion of Prostate Cancer Cells Through a LASS2/TMSG1-Independent Manner. Oncol Rep (2018) 39(1):298–306. doi: 10.3892/or.2017.6092 29138865

[115] Steffan JJ Dykes SS Coleman DT Adams LK Rogers D Carroll JL. Supporting a Role for the GTPase Rab7 in Prostate Cancer Progression. PLoS One (2014) 9(2):e87882. doi: 10.1371/journal.pone.0087882 24505328 PMC3914878

[116] Yang J Liu W Lu X Fu Y Li L Luo Y. High Expression of Small GTPase Rab3D Promotes Cancer Progression and Metastasis. Oncotarget (2015) 6(13):11125–38. doi: 10.18632/oncotarget.3575 PMC4484444 25823663

[117] Tan PY Chang CW Chng KR Wansa KD Sung WK Cheung E. Integration of Regulatory Networks by NKX3-1 Promotes Androgen-Dependent Prostate Cancer Survival. Mol Cell Biol (2012) 32(2):399–414. doi: 10.1128/MCB.05958-11 22083957 PMC3255774

[118] Luo ML Gong C Chen CH Hu H Huang P Zheng M. The Rab2A GTPase Promotes Breast Cancer Stem Cells and Tumorigenesis *via* Erk Signaling Activation. Cell Rep (2015) 11(1):111–24. doi: 10.1016/j.celrep.2015.03.002 PMC4401741 25818297

[119] Riplinger SM Wabnitz GH Kirchgessner H Jahraus B Lasitschka F Schulte B. Metastasis of Prostate Cancer and Melanoma Cells in a Preclinical *In Vivo* Mouse Model is Enhanced by L-Plastin Expression and Phosphorylation. Mol Cancer (2014) 13:10. doi: 10.1186/1476-4598-13-10 24438191 PMC3899628

[120] Colaço A Jäättelä M. Ragulator-A Multifaceted Regulator of Lysosomal Signaling and Trafficking. J Cell Biol (2017) 216(12):3895–8. doi: 10.1083/jcb.201710039 PMC5716293 29138253

[121] Eriksson P Aine M Veerla S Liedberg F Sjödahl G Höglund M. Molecular Subtypes of Urothelial Carcinoma are Defined by Specific Gene Regulatory Systems. BMC Med Genomics (2015) 8:25. doi: 10.1186/s12920-015-0101-5 26008846 PMC4446831

[122] Chang SS. Overview of Prostate-Specific Membrane Antigen. Rev Urol (2004) 6 Suppl 10:S13–8. PMC1472940 16985927

[123] Gu TL Cherry J Tucker M Wu J Reeves C Polakiewicz RD. Identification of Activated Tnk1 Kinase in Hodgkin's Lymphoma. Leukemia (2010) 24(4):861–5. doi: 10.1038/leu.2009.293 20090780

[124] Hazarika P McCarty MF Prieto VG George S Babu D Koul D. Up-Regulation of Flotillin-2 is Associated With Melanoma Progression and Modulates Expression of the Thrombin Receptor Protease Activated Receptor 1. Cancer Res (2004) 64(20):7361–9. doi: 10.1158/0008-5472.CAN-04-0823 15492257

[125] Liu J Huang W Ren C Wen Q Liu W Yang X. Flotillin-2 Promotes Metastasis of Nasopharyngeal Carcinoma by Activating NF-κb and PI3K/Akt3 Signaling Pathways. Sci Rep (2015) 5:11614. doi: 10.1038/srep11614 26206082 PMC4648439

[126] Wang CH Zhu XD Ma DN Sun HC Gao DM Zhang N. Flot2 Promotes Tumor Growth and Metastasis Through Modulating Cell Cycle and Inducing Epithelial-Mesenchymal Transition of Hepatocellular Carcinoma. Am J Cancer Res (2017) 7(5):1068–83. PMC5446475 28560058

[127] Ronquist KG Ronquist G Larsson A Carlsson L. Proteomic Analysis of Prostate Cancer Metastasis-Derived Prostasomes. Anticancer Res (2010) 30(2):285–90. 20332430

[128] Kennedy L Sandhu JK Harper ME Cuperlovic-Culf M. Role of Glutathione in Cancer: From Mechanisms to Therapies. Biomolecules (2020) 10(10):1429. doi: 10.3390/biom10101429 PMC7600400 33050144

[129] Zhao X Fu J Du J Xu W. The Role of D-3-Phosphoglycerate Dehydrogenase in Cancer. Int J Biol Sci (2020) 16(9):1495–506. doi: 10.7150/ijbs.41051 PMC7097917 32226297

[130] Kim E Kwak H Ahn K. Cytosolic Aminopeptidases Influence MHC Class I-Mediated Antigen Presentation in an Allele-Dependent Manner. J Immunol (Baltimore Md. 1950) (2009) 183(11):7379–87. doi: 10.4049/jimmunol.0901489 19917696

[131] Almaguel FA Sanchez TW Ortiz-Hernandez GL Casiano CA. Alpha-Enolase: Emerging Tumor-Associated Antigen, Cancer Biomarker, and Oncotherapeutic Target. Front Genet (2020) 11:614726. doi: 10.3389/fgene.2020.614726 33584813 PMC7876367

[132] Chen J Cheng X Merched-Sauvage M Caulin C Roop DR Koch PJ. An Unexpected Role for Keratin 10 End Domains in Susceptibility to Skin Cancer. J Cell Sci (2006) 119(Pt 24):5067–76. doi: 10.1242/jcs.03298 17118961

[133] Guo C Liu S Wang J Sun MZ Greenaway FT. ACTB in Cancer. Clin Chim acta; Int J Clin Chem (2013) 417:39–44. doi: 10.1016/j.cca.2012.12.012 23266771

[134] Calvert AE Chalastanis A Wu Y Hurley LA Kouri FM Bi Y. Cancer-Associated IDH1 Promotes Growth and Resistance to Targeted Therapies in the Absence of Mutation. Cell Rep (2017) 19(9):1858–73. doi: 10.1016/j.celrep.2017.05.014 PMC5564207 28564604

[135] Jelski W Szmitkowski M. Alcohol Dehydrogenase (ADH) and Aldehyde Dehydrogenase (ALDH) in the Cancer Diseases. Clin Chim acta; Int J Clin Chem (2008) 395(1-2):1–5. doi: 10.1016/j.cca.2008.05.001 18505683

[136] Bardella C Pollard PJ Tomlinson I. SDH Mutations in Cancer. Biochim Biophys Acta (2011) 1807(11):1432–43. doi: 10.1016/j.bbabio.2011.07.003 21771581

[137] Huang D Cao L Zheng S. CAPZA1 Modulates EMT by Regulating Actin Cytoskeleton Remodelling in Hepatocellular Carcinoma. J Exp Clin Cancer Res CR (2017) 36(1):13. doi: 10.1186/s13046-016-0474-0 28093067 PMC5240199

[138] Hulin JA Tommasi S Elliot D Mangoni AA. Small Molecule Inhibition of DDAH1 Significantly Attenuates Triple Negative Breast Cancer Cell Vasculogenic Mimicry *In Vitro* . Biomed Pharmacother (2019) 111:602–12. doi: 10.1016/j.biopha.2018.12.117 30611984

[139] Scannell M Flanagan MB deStefani A Wynne KJ Cagney G Godson C. Annexin-1 and Peptide Derivatives are Released by Apoptotic Cells and Stimulate Phagocytosis of Apoptotic Neutrophils by Macrophages. J Immunol (Baltimore Md. 1950) (2007) 178(7):4595–605. doi: 10.4049/jimmunol.178.7.4595 17372018

[140] Blume KE Soeroes S Waibel M Keppeler H Wesselborg S Herrmann M. Cell Surface Externalization of Annexin A1 as a Failsafe Mechanism Preventing Inflammatory Responses During Secondary Necrosis. J Immunol (Baltimore Md. 1950) (2009) 183(12):8138–47. doi: 10.4049/jimmunol.0902250 20007579

[141] Huggins A Paschalidis N Flower RJ Perretti M D'Acquisto F. Annexin-1-Deficient Dendritic Cells Acquire a Mature Phenotype During Differentiation. FASEB J Off Publ Fed Am Soc Exp Biol (2009) 23(4):985–96. doi: 10.1096/fj.08-119040 19029200

[142] Bist P Shu S Lee H Arora S Nair S Lim JY. Annexin-A1 Regulates TLR-Mediated IFN-β Production Through an Interaction With TANK-Binding Kinase 1. J Immunol (Baltimore Md. 1950) (2013) 191(8):4375–82. doi: 10.4049/jimmunol.1301504 24048896

[143] Jorge YC Mataruco MM Araújo LP Rossi AF de Oliveira JG Valsechi MC. Expression of Annexin-A1 and Galectin-1 Anti-Inflammatory Proteins and mRNA in Chronic Gastritis and Gastric Cancer. Mediators Inflammation (2013) 2013:152860. doi: 10.1155/2013/152860 PMC3574744 23431236

[144] Ullah MF. Sulforaphane (SFN): An Isothiocyanate in a Cancer Chemoprevention Paradigm. Medicines (Basel Switzerland) (2015) 2(3):141–56. doi: 10.3390/medicines2030141 PMC5456215 28930206

[145] Tang J Qin Z Han P Wang W Yang C Xu Z. High Annexin A5 Expression Promotes Tumor Progression and Poor Prognosis in Renal Cell Carcinoma. Int J Oncol (2017) 50(5):1839–47. doi: 10.3892/ijo.2017.3942 28393205

[146] Yang L Lu P Yang X Li K Qu S. Annexin A3, A Calcium-Dependent Phospholipid-Binding Protein: Implication in Cancer. Front Mol Biosci (2021) 8:716415. doi: 10.3389/fmolb.2021.716415 34355022 PMC8329414

[147] Iwamoto K Takahashi H Okuzaki D Osawa H Ogino T Miyoshi N. Syntenin-1 Promotes Colorectal Cancer Stem Cell Expansion and Chemoresistance by Regulating Prostaglandin E2 Receptor. Br J Cancer (2020) 123(6):955–64. doi: 10.1038/s41416-020-0965-9 PMC7492211 32595209

[148] van Ommeren R Staudt MD Xu H Hebb MO. Advances in HSP27 and HSP90-Targeting Strategies for Glioblastoma. J Neuro-oncol (2016) 127(2):209–19. doi: 10.1007/s11060-016-2070-8 26842818

[149] Hansen RK Parra I Hilsenbeck SG Himelstein B Fuqua SA. Hsp27-Induced MMP-9 Expression is Influenced by the Src Tyrosine Protein Kinase Yes. Biochem Biophys Res Commun (2001) 282(1):186–93. doi: 10.1006/bbrc.2001.4548 11263990

[150] Lemieux P Oesterreich S Lawrence JA Steeg PS Hilsenbeck SG Harvey JM. The Small Heat Shock Protein Hsp27 Increases Invasiveness But Decreases Motility of Breast Cancer Cells. Invasion Metastasis (1997) 17(3):113–23. 9702938

[151] Liang HH Huang CY Chou CW Makondi PT Huang MT Wei PL. Heat Shock Protein 27 Influences the Anti-Cancer Effect of Curcumin in Colon Cancer Cells Through ROS Production and Autophagy Activation. Life Sci (2018) 209:43–51. doi: 10.1016/j.lfs.2018.07.047 30056019

[152] Chang X-Z Li D-Q Hou Y-F Wu J Lu J-S Di G-H. Identification of the Functional Role of Peroxiredoxin 6 in the Progression of Breast Cancer. Breast Cancer Res (2007) 9(6):1–15. doi: 10.1186/bcr1789 PMC2246172 17980029

[153] Chen T Huang Z Tian Y Lin B He R Wang H. Clinical Significance and Prognostic Value of Triosephosphate Isomerase Expression in Gastric Cancer. Medicine (2017) 96(19):e6865. doi: 10.1097/MD.0000000000006865 28489783 PMC5428617

[154] Schoentgen F Jonic SJ APA. PEBP1/RKIP: From Multiple Functions to a Common Role in Cellular Processes. (2018) arXiv:1802.02378. doi: doi: 10.48550/arXiv.1802.02378

[155] Ishiguro H Izumi K Kashiwagi E Zheng Y Li Y Kawahara T. Semenogelin I Promotes Prostate Cancer Cell Growth *via* Functioning as an Androgen Receptor Coactivator and Protecting Against Zinc Cytotoxicity. Am J Cancer Res (2015) 5(2):738–47. PMC4396034 25973311

[156] Hileman EA Achanta G Huang P. Superoxide Dismutase: An Emerging Target for Cancer Therapeutics. Expert Opin Ther Targets (2001) 5(6):697–710. doi: 10.1517/14728222.5.6.697 12540279

[157] Hosseini SM Okoye I Chaleshtari MG Hazhirkarzar B Mohamadnejad J Azizi G. E2 Ubiquitin-Conjugating Enzymes in Cancer: Implications for Immunotherapeutic Interventions. Clin Chim acta; Int J Clin Chem (2019) 498:126–34. doi: 10.1016/j.cca.2019.08.020 31445029

[158] Edechi CA Ikeogu NM Akaluka GN Terceiro LEL Machado M Salako ES. The Prolactin Inducible Protein Modulates Antitumor Immune Responses and Metastasis in a Mouse Model of Triple Negative Breast Cancer. Front Oncol (2021) 11:639859. doi: 10.3389/fonc.2021.639859 33777801 PMC7994859

[159] Markowitz J Carson WE. 3rd, Review of S100A9 Biology and its Role in Cancer. Biochim Biophys Acta (2013) 1835(1):100–9. doi: 10.1016/j.bbcan.2012.10.003 PMC3670606 23123827

[160] Wei X Zhou J Hong L Xu Z Zhao H Wu X. Hint1 Expression Inhibits Proliferation and Promotes Radiosensitivity of Human SGC7901 Gastric Cancer Cells. Oncol Lett (2018) 16(2):2135–42. doi: 10.3892/ol.2018.8900 PMC6036515 30008911

[161] Duman C Yaqubi K Hoffmann A Acikgöz AA Korshunov A Bendszus M. Acyl-CoA-Binding Protein Drives Glioblastoma Tumorigenesis by Sustaining Fatty Acid Oxidation. Cell Metab (2019) 30(2):274–89.e5. doi: 10.1016/j.cmet.2019.04.004 31056285

[162] Meng M Sang L Wang X. S100 Calcium Binding Protein A11 (S100A11) Promotes The Proliferation, Migration And Invasion Of Cervical Cancer Cells, And Activates Wnt/β-Catenin Signaling. Onco Targets Ther (2019) 12:8675–85. doi: 10.2147/OTT.S225248 PMC6815786 31695426

[163] Luanpitpong S Talbott SJ Rojanasakul Y Nimmannit U Pongrakhananon V Wang L. Regulation of Lung Cancer Cell Migration and Invasion by Reactive Oxygen Species and Caveolin-1. J Biol Chem (2010) 285(50):38832–40. doi: 10.1074/jbc.M110.124958 PMC2998081 20923773

[164] Basu GD Azorsa DO Kiefer JA Rojas AM Tuzmen S Barrett MT. Functional Evidence Implicating S100P in Prostate Cancer Progression. Int J Cancer (2008) 123(2):330–9. doi: 10.1002/ijc.23447 18452169

[165] Lange I Geerts D Feith DJ Mocz G Koster J Bachmann AS. Novel Interaction of Ornithine Decarboxylase With Sepiapterin Reductase Regulates Neuroblastoma Cell Proliferation. J Mol Biol (2014) 426(2):332–46. doi: 10.1016/j.jmb.2013.09.037 PMC4013099 24096079

[166] Liu H Liu JY Wu X Zhang JT. Biochemistry, Molecular Biology, and Pharmacology of Fatty Acid Synthase, an Emerging Therapeutic Target and Diagnosis/Prognosis Marker. Int J Biochem Mol Biol (2010) 1(1):69–89. 20706604 PMC2919769

[167] Słomnicki ŁP Nawrot B Leśniak W. S100A6 Binds P53 and Affects its Activity. Int J Biochem Cell Biol (2009) 41(4):784–90. doi: 10.1016/j.biocel.2008.08.007 18765292

[168] van Dieck J Brandt T Teufel DP Veprintsev DB Joerger AC Fersht AR. Molecular Basis of S100 Proteins Interacting With the P53 Homologs P63 and P73. Oncogene (2010) 29(14):2024–35. doi: 10.1038/onc.2009.490 20140014

[169] Qin DN Zhu JG Ji CB Chunmei S Kou CZ Zhu GZ. Monoclonal Antibody to Six Transmembrane Epithelial Antigen of Prostate-4 Influences Insulin Sensitivity by Attenuating Phosphorylation of P13K (P85) and Akt: Possible Mitochondrial Mechanism. J Bioenerg Biomembr (2011) 43(3):247–55. doi: 10.1007/s10863-011-9360-9 21647634

[170] Webber JP Spary LK Sanders AJ Chowdhury R Jiang WG Steadman R. Differentiation of Tumour-Promoting Stromal Myofibroblasts by Cancer Exosomes. Oncogene (2015) 34(3):290–302. doi: 10.1038/onc.2013.560 24441045

[171] Hayes JD Dinkova-Kostova AT Tew KD. Oxidative Stress in Cancer. Cancer Cell (2020) 38(2):167–97. doi: 10.1016/j.ccell.2020.06.001 PMC7439808 32649885

[172] Ying W. NAD+/NADH and NADP+/NADPH in Cellular Functions and Cell Death: Regulation and Biological Consequences. Antioxid Redox Signaling (2008) 10(2):179–206. doi: 10.1089/ars.2007.1672 18020963

[173] Neophytou CM Panagi M Stylianopoulos T Papageorgis P. The Role of Tumor Microenvironment in Cancer Metastasis: Molecular Mechanisms and Therapeutic Opportunities. Cancers (2021) 13(9):2053. doi: 10.3390/cancers13092053 33922795 PMC8122975

[174] Thuringer D Jego G Wettstein G Terrier O Cronier L Yousfi N. Extracellular HSP27 Mediates Angiogenesis Through Toll-Like Receptor 3. FASEB J Off Publ Fed Am Soc Exp Biol (2013) 27(10):4169–83. doi: 10.1096/fj.12-226977 23804239

[175] Jang D Kwon H Jeong K Lee J Pak Y. Essential Role of Flotillin-1 Palmitoylation in the Intracellular Localization and Signaling Function of IGF-1 Receptor. J Cell Sci (2015) 128(11):2179–90. doi: 10.1242/jcs.169409 25908865

[176] Shinbo Y Taira T Niki T Iguchi-Ariga SM Ariga H. DJ-1 Restores P53 Transcription Activity Inhibited by Topors/P53bp3. Int J Oncol (2005) 26(3):641–8. doi: 10.3892/ijo.26.3.641 15703819

[177] Takahashi-Niki K Ganaha Y Niki T Nakagawa S Kato-Ose I Iguchi-Ariga SMM. DJ-1 Activates SIRT1 Through its Direct Binding to SIRT1. Biochem Biophys Res Commun (2016) 474(1):131–6. doi: 10.1016/j.bbrc.2016.04.084 27105916

[178] Watanabe J Kamata Y Seo N Okayasu I Kuramoto H. Stimulatory Effect of Estrogen on the Growth of Endometrial Cancer Cells is Regulated by Cell-Cycle Regulators. J Steroid Biochem Mol Biol (2007) 107(3-5):163–71. doi: 10.1016/j.jsbmb.2007.03.045 17681750

[179] Bosch M Gil J Bachs O Agell N. Calmodulin Inhibitor W13 Induces Sustained Activation of ERK2 and Expression of P21(Cip1). J Biol Chem (1998) 273(34):22145–50. doi: 10.1074/jbc.273.34.22145 9705360

[180] Pandey MK Prasad S Tyagi AK Deb L Huang J Karelia DN. Targeting Cell Survival Proteins for Cancer Cell Death. Pharmaceut (Basel Switzerland) (2016) 9(1):11. doi: 10.3390/ph9010011 PMC4812375 26927133

[181] Boroughs LK DeBerardinis RJ. Metabolic Pathways Promoting Cancer Cell Survival and Growth. Nat Cell Biol (2015) 17(4):351–9. doi: 10.1038/ncb3124 PMC4939711 25774832

[182] Liu Y Shi K Chen Y Wu X Chen Z Cao K. Exosomes and Their Role in Cancer Progression. Front Oncol (2021) 11:639159. doi: 10.3389/fonc.2021.639159 33828985 PMC8020998

[183] Gordon SR Maute RL Dulken BW Hutter G George BM McCracken MN. PD-1 Expression by Tumour-Associated Macrophages Inhibits Phagocytosis and Tumour Immunity. Nature (2017) 545(7655):495–9. doi: 10.1038/nature22396 PMC5931375 28514441

[184] Dong H Strome SE Salomao DR Tamura H Hirano F Flies DB. Tumor-Associated B7-H1 Promotes T-Cell Apoptosis: A Potential Mechanism of Immune Evasion. Nat Med (2002) 8(8):793–800. doi: 10.1038/nm730 12091876

[185] Clayton A Mitchell JP Court J Linnane S Mason MD Tabi Z. Human Tumor-Derived Exosomes Down-Modulate NKG2D Expression. J Immunol (Baltimore Md. 1950) (2008) 180(11):7249–58. doi: 10.4049/jimmunol.180.11.7249 18490724

[186] Clayton A Mitchell JP Court J Mason MD Tabi Z. Human Tumor-Derived Exosomes Selectively Impair Lymphocyte Responses to Interleukin-2. Cancer Res (2007) 67(15):7458–66. doi: 10.1158/0008-5472.CAN-06-3456 17671216

[187] Novitskiy SV Ryzhov S Zaynagetdinov R Goldstein AE Huang Y Tikhomirov OY. Adenosine Receptors in Regulation of Dendritic Cell Differentiation and Function. Blood (2008) 112(5):1822–31. doi: 10.1182/blood-2008-02-136325 PMC2518889 18559975

[188] Wang R Liu Y Liu L Chen M Wang X Yang J. Tumor Cells Induce LAMP2a Expression in Tumor-Associated Macrophage for Cancer Progression. EBioMedicine (2019) 40:118–34. doi: 10.1016/j.ebiom.2019.01.045 PMC6413476 30711520

[189] Cho JA Park H Lim EH Lee KW. Exosomes From Breast Cancer Cells can Convert Adipose Tissue-Derived Mesenchymal Stem Cells Into Myofibroblast-Like Cells. Int J Oncol (2012) 40(1):130–8. doi: 10.3892/ijo.2011.1193 21904773

[190] Zhong H Chiles K Feldser D Laughner E Hanrahan C Georgescu MM. Modulation of Hypoxia-Inducible Factor 1alpha Expression by the Epidermal Growth Factor/Phosphatidylinositol 3-Kinase/PTEN/AKT/FRAP Pathway in Human Prostate Cancer Cells: Implications for Tumor Angiogenesis and Therapeutics. Cancer Res (2000) 60(6):1541–5. 10749120

[191] Chien CH Lee MJ Liou HC Liou HH Fu WM. Local Immunosuppressive Microenvironment Enhances Migration of Melanoma Cells to Lungs in DJ-1 Knockout Mice. PLoS One (2015) 10(2):e0115827. doi: 10.1371/journal.pone.0115827 25706411 PMC4338246

[192] Huggins C Hodges CV. Studies on Prostatic Cancer. I. The Effect of Castration, of Estrogen and Androgen Injection on Serum Phosphatases in Metastatic Carcinoma of the Prostate. CA: Cancer J Clin (1972) 22(4):232–40. doi: 10.3322/canjclin.22.4.232 4625049

[193] Attard G Reid AH Auchus RJ Hughes BA Cassidy AM Thompson E. Clinical and Biochemical Consequences of CYP17A1 Inhibition With Abiraterone Given With and Without Exogenous Glucocorticoids in Castrate Men With Advanced Prostate Cancer. J Clin Endocrinol Metab (2012) 97(2):507–16. doi: 10.1210/jc.2011-2189 22170708

[194] Cheng KW Agarwal R Mitra S Lee JS Carey M Gray JW. Rab25 Increases Cellular ATP and Glycogen Stores Protecting Cancer Cells From Bioenergetic Stress. EMBO Mol Med (2012) 4(2):125–41. doi: 10.1002/emmm.201100193 PMC3306554 22253197

[195] Walther TC Chung J Farese RV Jrs. Lipid Droplet Biogenesis. Annu Rev Cell Dev Biol (2017) 33:491–510. doi: 10.1146/annurev-cellbio-100616-060608 28793795 PMC6986389

[196] Wu L Xu D Zhou L Xie B Yu L Yang H. Rab8a-AS160-MSS4 Regulatory Circuit Controls Lipid Droplet Fusion and Growth. Dev Cell (2014) 30(4):378–93. doi: 10.1016/j.devcel.2014.07.005 25158853

[197] Raposo G Stoorvogel W. Extracellular Vesicles: Exosomes, Microvesicles, and Friends. J Cell Biol (2013) 200(4):373–83. doi: 10.1083/jcb.201211138 PMC3575529 23420871

[198] Schiefermeier N Scheffler JM de Araujo ME Stasyk T Yordanov T Ebner HL. The Late Endosomal P14-MP1 (LAMTOR2/3) Complex Regulates Focal Adhesion Dynamics During Cell Migration. J Cell Biol (2014) 205(4):525–40. doi: 10.1083/jcb.201310043 PMC4033770 24841562

[199] Tuli A Thiery J James AM Michelet X Sharma M Garg S. Arf-Like GTPase Arl8b Regulates Lytic Granule Polarization and Natural Killer Cell-Mediated Cytotoxicity. Mol Biol Cell (2013) 24(23):3721–35. doi: 10.1091/mbc.e13-05-0259 PMC3842998 24088571

[200] Shin IY Sung NY Lee YS Kwon TS Si Y Lee YS. The Expression of Multiple Proteins as Prognostic Factors in Colorectal Cancer: Cathepsin D, P53, COX-2, Epidermal Growth Factor Receptor, C-erbB-2, and Ki-67. Gut Liver (2014) 8(1):13–23. doi: 10.5009/gnl.2014.8.1.13 24516696 PMC3916682

[201] Hahm ER Singh KB Kim SH Powolny AA Singh SV. The Role of Lysosome-Associated Membrane Protein 2 in Prostate Cancer Chemopreventive Mechanisms of Sulforaphane. Cancer Prev Res (Phila) (2020) 13(8):661–72. doi: 10.1158/1940-6207.CAPR-20-0054 PMC7415716 32434809

[202] Ding ZB Fu XT Shi YH Zhou J Peng YF Liu WR. Lamp2a is Required for Tumor Growth and Promotes Tumor Recurrence of Hepatocellular Carcinoma. Int J Oncol (2016) 49(6):2367–76. doi: 10.3892/ijo.2016.3754 27840904

[203] Timme TL Goltsov A Tahir S Li L Wang J Ren C. Caveolin-1 is Regulated by C-Myc and Suppresses C-Myc-Induced Apoptosis. Oncogene (2000) 19(29):3256–65. doi: 10.1038/sj.onc.1203654 10918582

[204] Sporn MB. The War on Cancer. Lancet (London England) (1996) 347(9012):1377–81. doi: 10.1016/S0140-6736(96)91015-6 8637346

[205] Bijnsdorp IV Geldof AA Lavaei M Piersma SR van Moorselaar RJ Jimenez CR. Exosomal ITGA3 Interferes With non-Cancerous Prostate Cell Functions and is Increased in Urine Exosomes of Metastatic Prostate Cancer Patients. J Extracell Vesicles (2013) 2:22079. doi: 10.3402/jev.v2i0.22097 PMC3873120 24371517

[206] Allard D Chrobak P Allard B Messaoudi N Stagg J. Targeting the CD73-Adenosine Axis in Immuno-Oncology. Immunol Lett (2019) 205:31–9. doi: 10.1016/j.imlet.2018.05.001 29758241

[207] Ebos JM Lee CR Cruz-Munoz W Bjarnason GA Christensen JG Kerbel RS. Accelerated Metastasis After Short-Term Treatment With a Potent Inhibitor of Tumor Angiogenesis. Cancer Cell (2009) 15(3):232–9. doi: 10.1016/j.ccr.2009.01.021 PMC4540346 19249681

[208] Gesierich S Berezovskiy I Ryschich E Zöller M. Systemic Induction of the Angiogenesis Switch by the Tetraspanin D6.1A/CO-029. Cancer Res (2006) 66(14):7083–94. doi: 10.1158/0008-5472.CAN-06-0391 16849554

[209] Chen C Duckworth CA Zhao Q Pritchard DM Rhodes JM Yu LG. Increased Circulation of Galectin-3 in Cancer Induces Secretion of Metastasis-Promoting Cytokines From Blood Vascular Endothelium. Clin Cancer Res an Off J Am Assoc Cancer Res (2013) 19(7):1693–704. doi: 10.1158/1078-0432.CCR-12-2940 PMC3618858 23401226

[210] Zeng Q Li S Chepeha DB Giordano TJ Li J Zhang H. Crosstalk Between Tumor and Endothelial Cells Promotes Tumor Angiogenesis by MAPK Activation of Notch Signaling. Cancer Cell (2005) 8(1):13–23. doi: 10.1016/j.ccr.2005.06.004 16023595

[211] Casimiro S Guise TA Chirgwin J. The Critical Role of the Bone Microenvironment in Cancer Metastases. Mol Cell Endocrinol (2009) 310(1-2):71–81. doi: 10.1016/j.mce.2009.07.004 19616059

[212] Tauro BJ Mathias RA Greening DW Gopal SK Ji H Kapp EA. Oncogenic H-Ras Reprograms Madin-Darby Canine Kidney (MDCK) Cell-Derived Exosomal Proteins Following Epithelial-Mesenchymal Transition. Mol Cell Proteomics (2013) 12(8):2148–59. doi: 10.1074/mcp.M112.027086 PMC3734576 23645497

[213] Stuelten CH Parent CA Montell DJ. Cell Motility in Cancer Invasion and Metastasis: Insights From Simple Model Organisms. Nat Rev Cancer (2018) 18(5):296–312. doi: 10.1038/nrc.2018.15 29546880 PMC6790333

[214] Berditchevski F. Complexes of Tetraspanins With Integrins: More Than Meets the Eye. J Cell Sci (2001) 114(Pt 23):4143–51. doi: 10.1242/jcs.114.23.4143 11739647

[215] Yánez-Mó M Mittelbrunn M Sánchez-Madrid F. Tetraspanins and Intercellular Interactions. Microcirc (New York N.Y 1994) (2001) 8(3):153–68. doi: 10.1111/j.1549-8719.2001.tb00166.x 11498779

[216] Ang J Lijovic M Ashman LK Kan K Frauman AG. CD151 Protein Expression Predicts the Clinical Outcome of Low-Grade Primary Prostate Cancer Better Than Histologic Grading: A New Prognostic Indicator? Cancer Epidemiol Biomarkers Prev Publ Am Assoc Cancer Res Cosponsored by Am Soc Prev Oncol (2004) 13(11 Pt 1):1717–21. 15533898

[217] Detchokul S Williams ED Parker MW Frauman AG. Tetraspanins as Regulators of the Tumour Microenvironment: Implications for Metastasis and Therapeutic Strategies. Br J Pharmacol (2014) 171(24):5462–90. doi: 10.1111/bph.12260 PMC4290697 23731188

[218] Gesierich S Paret C Hildebrand D Weitz J Zgraggen K Schmitz-Winnenthal FH. Colocalization of the Tetraspanins, CO-029 and CD151, With Integrins in Human Pancreatic Adenocarcinoma: Impact on Cell Motility. Clin Cancer Res an Off J Am Assoc Cancer Res (2005) 11(8):2840–52. doi: 10.1158/1078-0432.CCR-04-1935 15837731

[219] Ang J Fang BL Ashman LK Frauman AG. The Migration and Invasion of Human Prostate Cancer Cell Lines Involves CD151 Expression. Oncol Rep (2010) 24(6):1593–7. doi: 10.3892/or_00001022 21042756

[220] He X Zheng Z Li J Ben Q Liu J Zhang J. DJ-1 Promotes Invasion and Metastasis of Pancreatic Cancer Cells by Activating SRC/ERK/uPA. Carcinogenesis (2012) 33(3):555–62. doi: 10.1093/carcin/bgs002 22223849

[221] Boscher C Nabi IR. Galectin-3- and Phospho-Caveolin-1-Dependent Outside-in Integrin Signaling Mediates the EGF Motogenic Response in Mammary Cancer Cells. Mol Biol Cell (2013) 24(13):2134–45. doi: 10.1091/mbc.e13-02-0095 PMC3694797 23657817

[222] Espinosa-Sánchez A Suárez-Martínez E Sánchez-Díaz L Carnero A. Therapeutic Targeting of Signaling Pathways Related to Cancer Stemness. Front Oncol (2020) 10:1533. doi: 10.3389/fonc.2020.01533 32984007 PMC7479251

[223] McCubrey JA Abrams SL Fitzgerald TL Cocco L Martelli AM Montalto G. Roles of Signaling Pathways in Drug Resistance, Cancer Initiating Cells and Cancer Progression and Metastasis. Adv Biol Regul (2015) 57:75–101. doi: 10.1016/j.jbior.2014.09.016 25453219

[224] Saha T. LAMP2A Overexpression in Breast Tumors Promotes Cancer Cell Survival *via* Chaperone-Mediated Autophagy. Autophagy (2012) 8(11):1643–56. doi: 10.4161/auto.21654 PMC3494593 22874552

[225] Bao L Lv L Feng J Chen Y Wang X Han S. miR-487b-5p Regulates Temozolomide Resistance of Lung Cancer Cells Through LAMP2-Medicated Autophagy. DNA Cell Biol (2016) 35(8):385–92. doi: 10.1089/dna.2016.3259 27097129

[226] Xu H Hong FZ Li S Zhang P Zhu L. Short Hairpin RNA-Mediated MDR1 Gene Silencing Increases Apoptosis of Human Ovarian Cancer Cell Line A2780/Taxol. Chin J Cancer Res (2012) 24(2):138–42. doi: 10.1007/s11670-012-0138-3 PMC3555269 23359770

[227] Stewart JJ White JT Yan X Collins S Drescher CW Urban ND. Proteins Associated With Cisplatin Resistance in Ovarian Cancer Cells Identified by Quantitative Proteomic Technology and Integrated With mRNA Expression Levels. Mol Cell Proteomics (2006) 5(3):433–43. doi: 10.1074/mcp.M500140-MCP200 16319398

